# Supplementary material for: Allogeneic iPSC-derived γδT cells demonstrate antitumor efficacy against patient-derived tissues
Source: Stem Cell Reports. 2026 Jul 23;21(8):103018. doi: 10.1016/j.stemcr.2026.103018 (PMC13476892; doi:10.1016/j.stemcr.2026.103018)
Supplement: Supplementary file 1 [file mmc1.pdf]

Stem Cell Reports, Volume *21*

Supplemental Information

Allogeneic iPSC-derived γδT cells demonstrate antitumor efficacy against patient-derived tissues

Ryoko Futai, Michiyo Koyanagi-Aoi, Aito Nakahashi, Daiki Katsura, Manabu Horikawa, Kazumasa Horie, Yoshihiro Kakeji, Yuzo Kodama, and Takashi Aoi

Figure S1

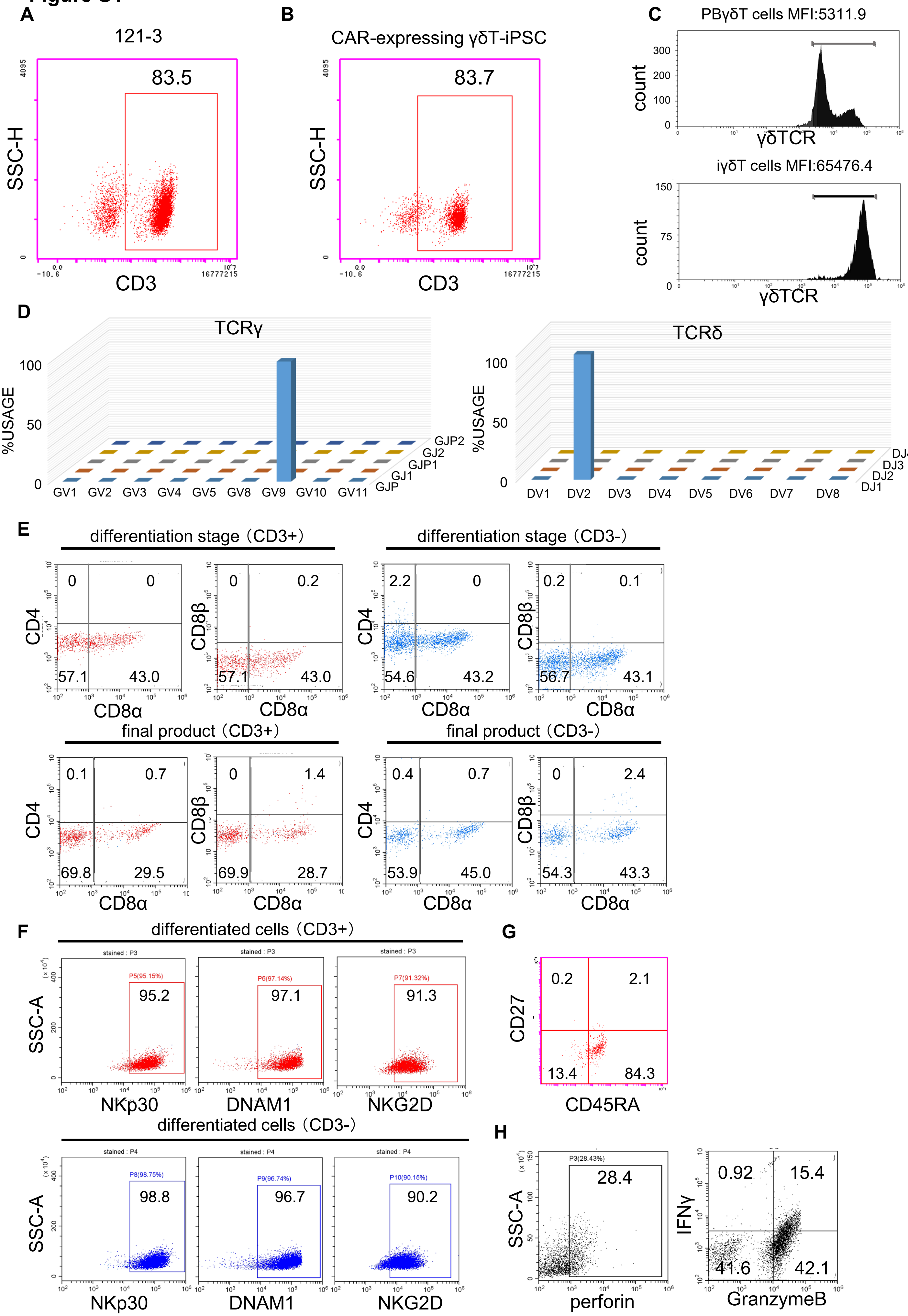
A
121-3
83.5
SSC-H
CD3
B
CAR-expressing γδT-iPSC
83.7
SSC-H
CD3
C
PBγδT cells MFI:5311.9
count
γδTCR
iγδT cells MFI:65476.4
count
γδTCR
D
TCRγ
%USAGE
GV1 GV2 GV3 GV4 GV5 GV8 GV9 GV10 GV11
GJP GJ1 GJP1 GJ2 GJP2
TCRδ
%USAGE
DV1 DV2 DV3 DV4 DV5 DV6 DV7 DV8
DJ1 DJ2 DJ3 DJ4
E
differentiation stage（CD3+）
differentiation stage（CD3-）
CD4
CD8β
CD8α
0 0 57.1 43.0
0 0.2 57.1 43.0
2.2 0 54.6 43.2
0.2 0.1 56.7 43.1
final product（CD3+）
final product（CD3-）
0.1 0.7 69.8 29.5
0 1.4 69.9 28.7
0.4 0.7 53.9 45.0
0 2.4 54.3 43.3
F
differentiated cells（CD3+）
SSC-A
95.2
97.1
91.3
NKp30
DNAM1
NKG2D
differentiated cells（CD3-）
SSC-A
98.8
96.7
90.2
NKp30
DNAM1
NKG2D
G
CD27
CD45RA
0.2 2.1 13.4 84.3
H
SSC-A
28.4
perforin
IFNγ
0.92 15.4 41.6 42.1
GranzymeB

Supplementary Figure 1. Phenotypic characterization and the functional profiling of iγδT cells differentiated from hiPSCs

A. A flow cytometry (FCM) analysis showing the percentage of CD3-positive iγδT cells differentiated from hiPSC clone 121-3.
B. FCM analysis showing the percentage of CD3-positive iγδT cells differentiated from the CAR-expressing γδT-iPSC line.
C. FCM analysis of the γδTCR expression in iγδ T cells and peripheral blood–derived γδ T cells (PBγδT cells). The iγδT cells exhibited a higher mean fluorescence intensity (MFI) with a single peak, in contrast to the bimodal distribution observed in the PBγδT cells.
D. Repertoire analysis of iγδT cells (derived from clone 121-3) at day 34 of differentiation. TCR repertoire profiling revealed predominant usage of TRGV9 and TRDV2. Amino acid sequence reads with a frequency of <1% were excluded, as such low-frequency reads may reflect sequencing errors.
E. FCM analysis of the CD4, CD8α, and CD8β expression in CD3-positive and CD3-negative iγδ T cells at the differentiation stage (day 26) and final product stage (day 58).
F. FCM analysis of the NKG2D, DNAM-1, and NKp30 expression in CD3-positive and CD3-negative iγδT cells at the final product stage, with >90% of the cells expressing these receptors.
G. FCM analysis of the CD45RA and CD27 expression in iγδ T cells, indicating a terminal effector phenotype (CD45RA+CD27-).
H. FCM analysis of the cytotoxic molecule expression. iγδ T cells were stimulated by co-culture with Jurkat cells at an E:T ratio of 2:1 for 6h in the presence of brefeldin A and produced IFN-γ , perforin, and granzyme B.

Figure S2

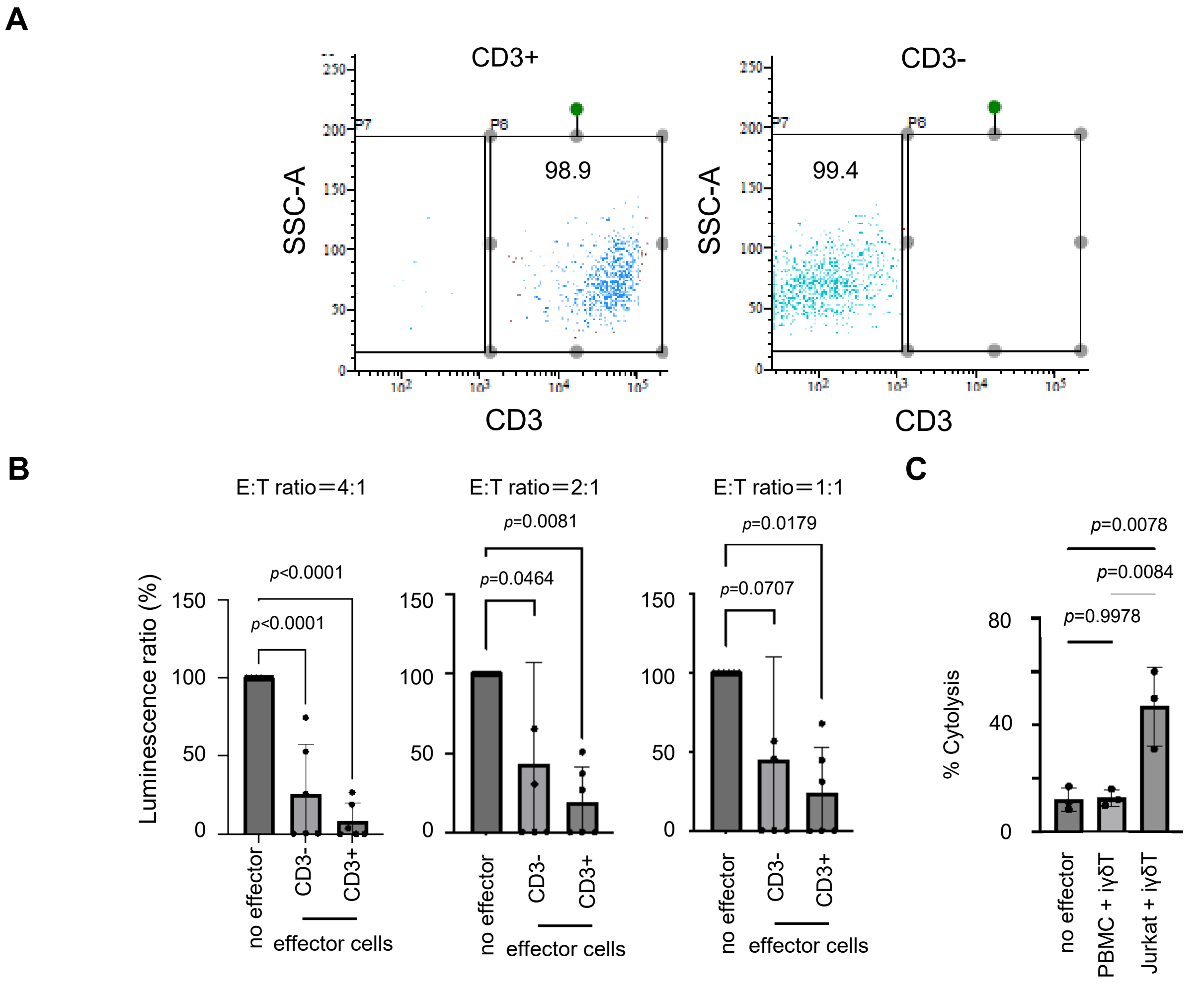

Supplementary Figure 2. Cytotoxic activity and safety profile of iγδT cells

A. A flow cytometry (FCM) analysis showing the sorting of iγδT cells into CD3-positive and CD3-negative cells.

B. The relative luminescence intensity of JurkatA containing CD3-positive cells and JurkatA containing CD3-negative cells, with the luminescence intensity of NC set to 100% (E:T ratios of 4:1, 2:1, and 1:1).

C. A mixed lymphocyte reaction (MLR) assay using PBMCs from three independent donors. The iγδT cells showed minimal cytotoxicity and a low alloreactivity toward PBMCs, whereas a robust cytotoxic activity was observed against Jurkat cells.

Figure S3

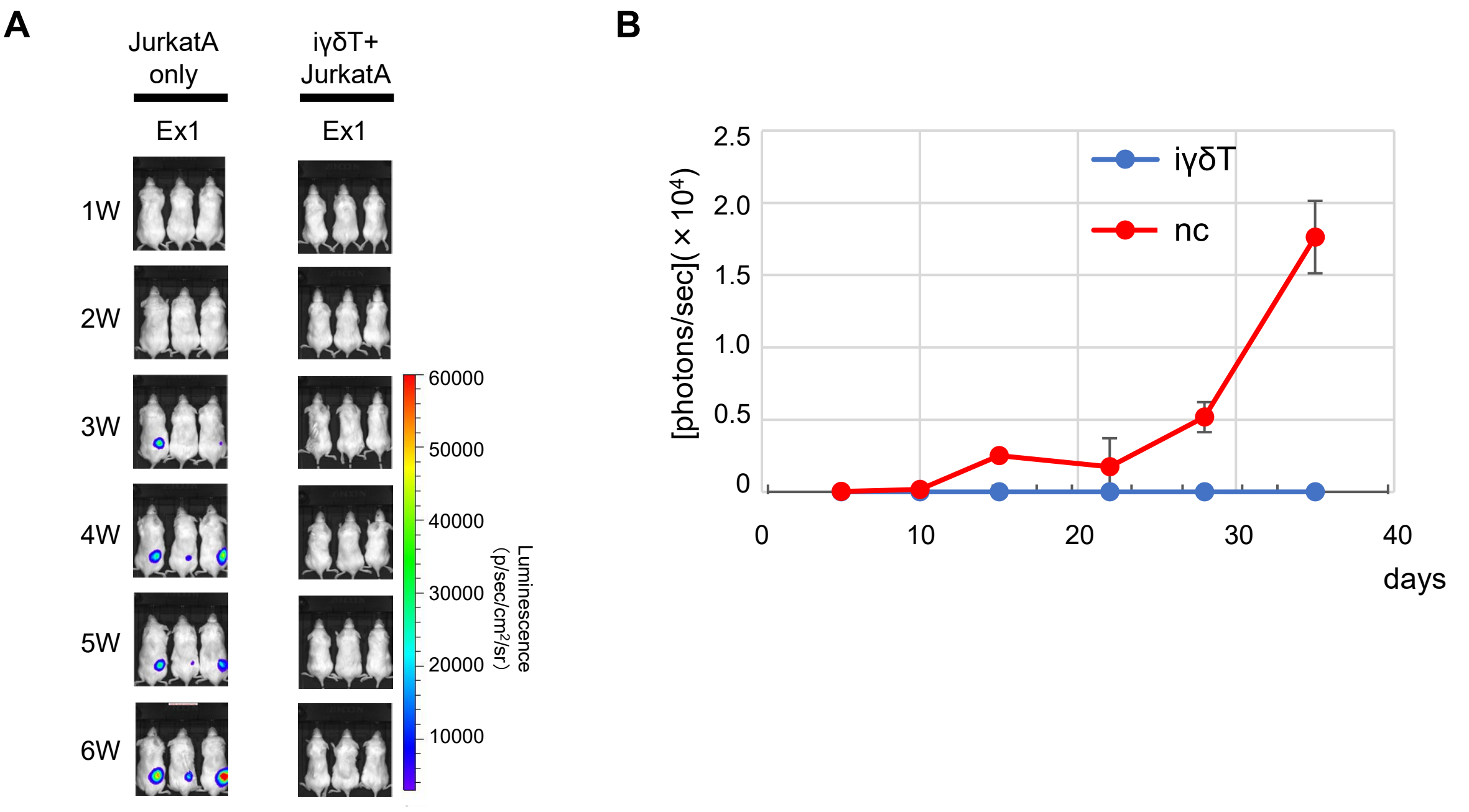

Supplementary Figure 3. In vivo antitumor activity of iγδT cells

A. Weekly bioluminescence images of the mice injected with JurkatA cells, with or without iγδT cell treatment, derived from a single differentiation batch (n = 3 per group) from the same lot (n = 3).

B. Quantification of the bioluminescence intensity of JurkatA tumors over time in mice. "nc" indicates"no effector control group." iγδT cell treatment resulted in a significant reduction in the tumor burden, with limited inter-animal variability.

Figure S4

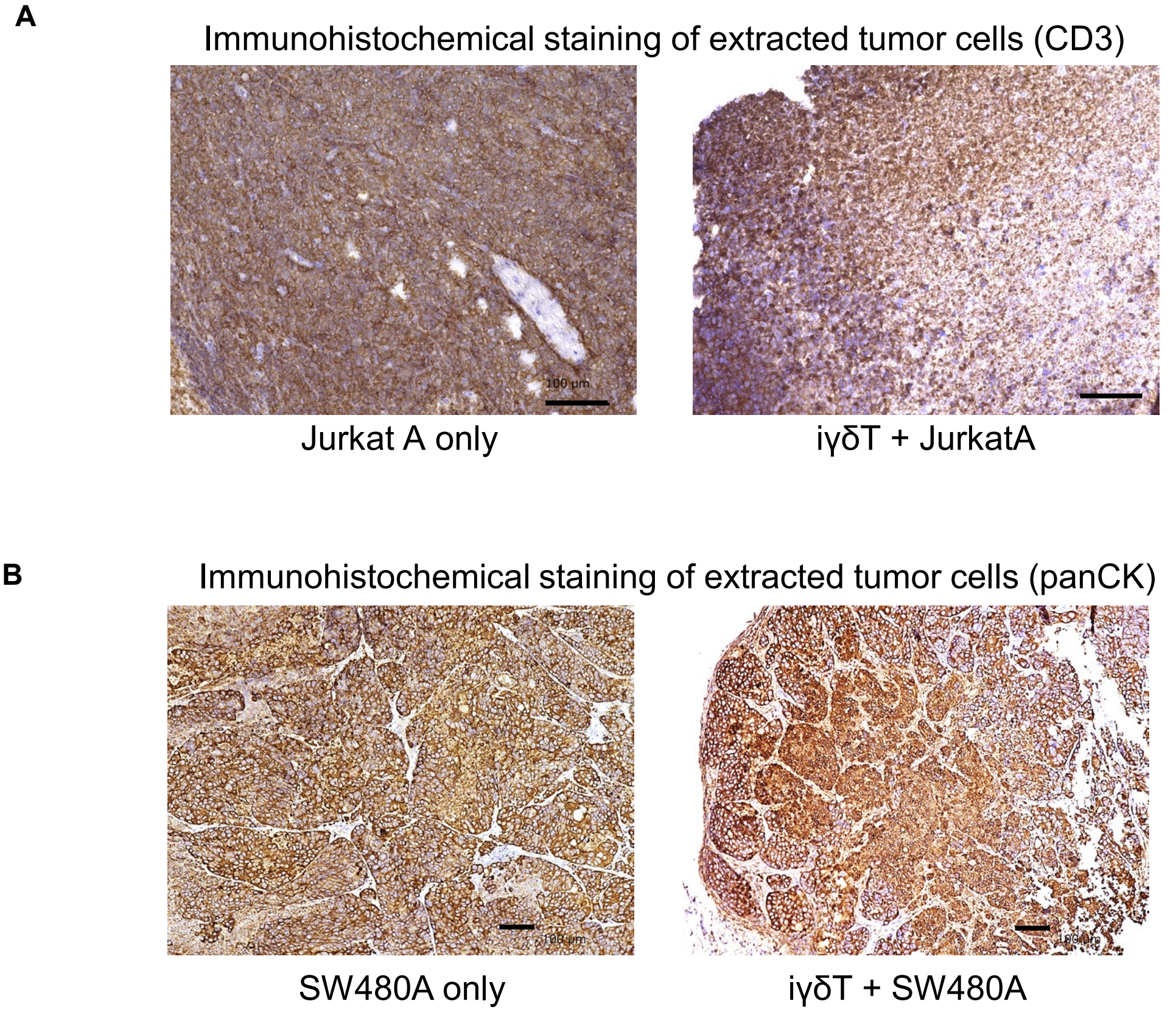

Supplementary Figure 4. Immunohistochemical Analysis of Tumor Tissues from JurkatA and SW480A Xenografts Following iγδT Cell Treatment
A. Left panel: CD3 immunohistochemical staining of tumor tissues extracted from JurkatA (NC group) transplanted into mice. Scale bars, 100 μm.
Right panel: CD3 immunohistochemical staining of tumor tissues extracted from JurkatA (iγδT group) transplanted into mice. Scale bars, 100 μm.
B. Left panel: panCK immunohistochemical staining of tumor tissues extracted from SW480A (NC-treated group) transplanted into mice. Scale bars, 100 μm.
Right panel: panCK immunohistochemical staining of tumor tissues extracted from SW480A (iγδT cell-treated group) transplanted into mice. Scale bars, 100 μm.

Figure S5

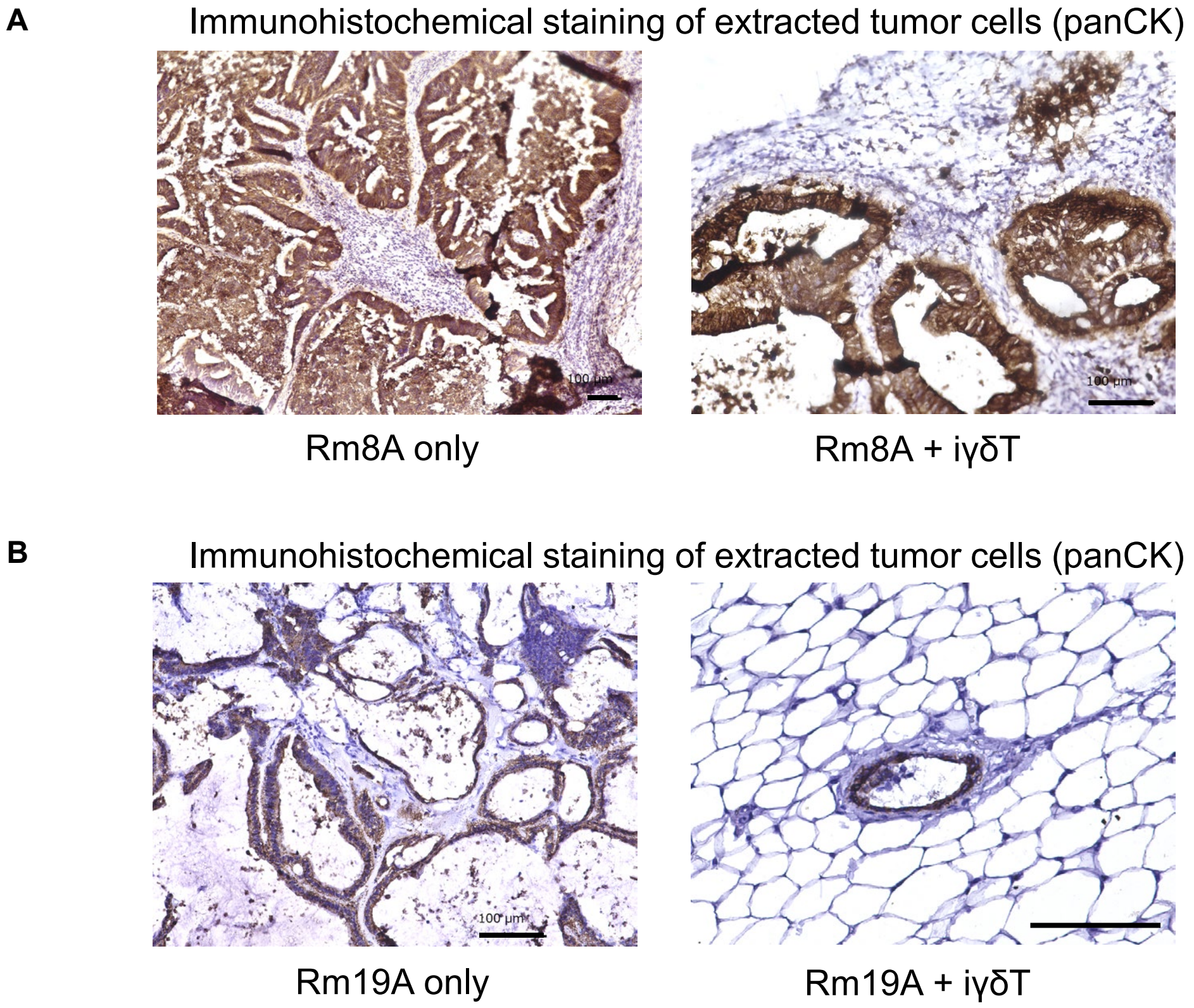

Supplementary Figure 5. panCK Immunohistochemical Staining of Rm8A and Rm19A Xenograft Tumors Following iγδT Cell Treatment

A. Left panel: panCK immunohistochemical staining of tumor tissues extracted from Rm8A (NC group) transplanted into mice. Scale bars, 100 µm.

Right panel: panCK immunohistochemical staining of tumor tissues extracted from Rm8A (iγδT cell locally treated group) transplanted into mice. Scale bars, 100 µm.

B. Left panel: panCK immunohistochemical staining of tumor tissues extracted from Rm19A (NC group) transplanted into mice. Scale bars, 100 µm.

Right panel: panCK immunohistochemical staining of tumor tissues extracted from Rm19A (iγδT cell-treated group) transplanted into mice. Scale bars, 50 µm.

Figure S6

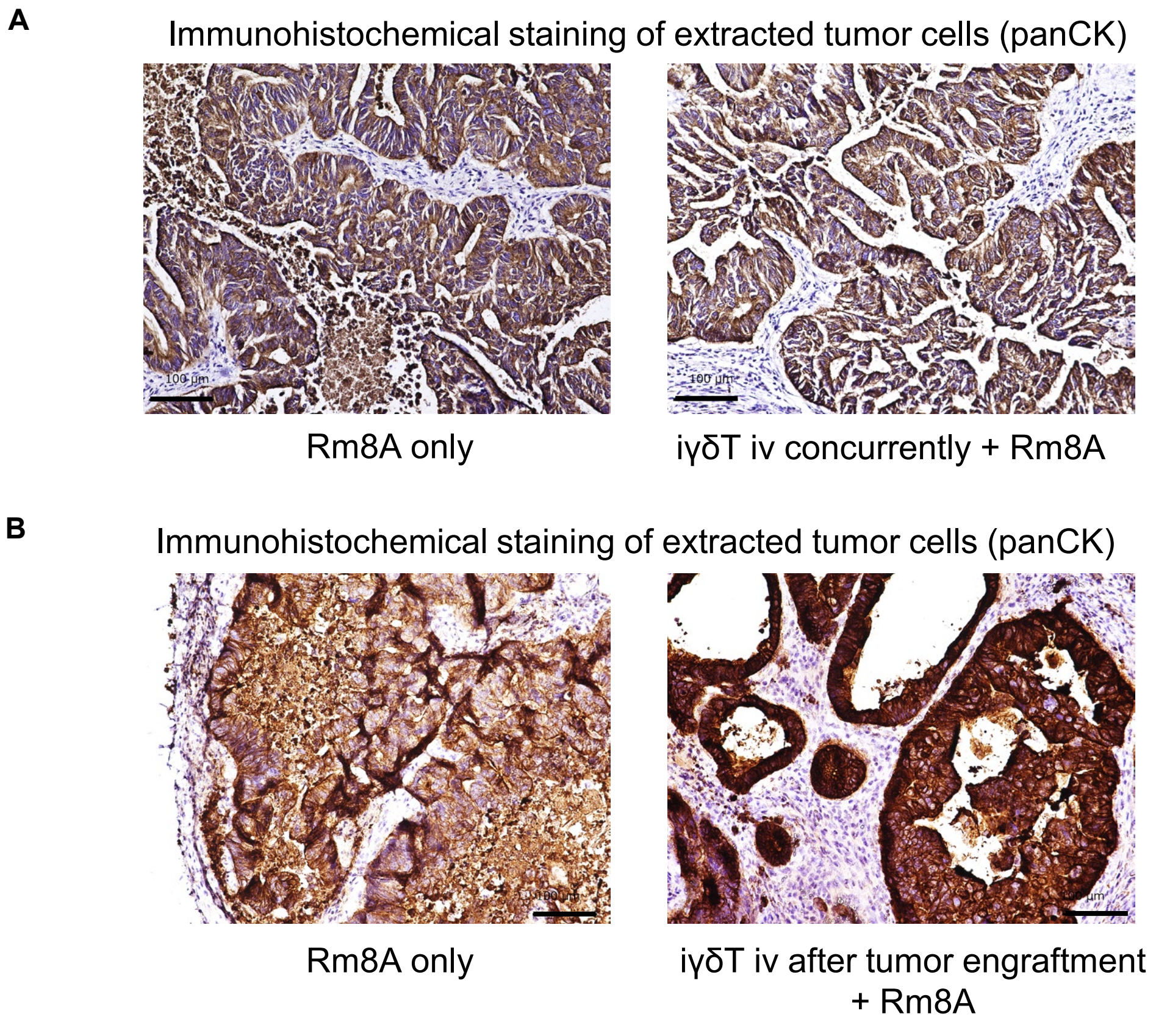

Supplementary Figure 6. panCK Immunohistochemical Staining of Rm8A Xenograft Tumors Following Intravenous iγδT Cell Administration

A. Left panel: panCK immunohistochemical staining of Rm8A (NC group) transplanted into mice. Scale bars, 100 µm.

Right panel: panCK immunohistochemical staining of Rm8A (iγδT cell intravenously co-administered group) transplanted into mice. Scale bars, 100 µm.

B. Left panel: panCK immunohistochemical staining of Rm8A (NC-treated group) transplanted into mice. Scale bars, 100 µm.

Right panel: panCK immunohistochemical staining of Rm8A (the group with iγδT cells intravenously administered after tumor engraftment) transplanted into mice. Scale bars, 100 µm.

Table S1

rank	TRGV	TRGJ	CDRJ	%reads
1	TRDV2	TRDJ1	CACDPLLGDTPRYTDKLIF	90.31884
2	TRDV2	TRDJ1	CACDPLLWDTPRYTDKLIF	2.062132
3	TRDV2	TRDJ1	CACDPLLVDTPRYTDKLIF	1.023523
4	TRDV2	TRDJ1	CACDPLLGDMPRYTDKLIF	0.327538
5	TRDV2	TRDJ1	CAFDPLLGDTPRYTDKLIF	0.31552
6	TRDV2	TRDJ1	CVCDPLLGDTPRYTDKLIF	0.282536
7	TRDV2	TRDJ1	CACDPLLGDTPRYTDKPIF	0.245717
8	TRDV2	TRDJ1	CAGDPLLGDTPRYTDKLIF	0.199437
9	TRDV2	TRDJ1	CACDPLLGETPRYTDKLIF	0.196369
10	TRDV2	TRDJ1	CACDPLLGDTPRYTEKLIF	0.178215
11	TRDV2	TRDJ1	CACDPLLGDTPRYTDKLTF	0.171312
12	TRDV2	TRDJ1	CACDPLLGDTPRYTNKLIF	0.170289
13	TRDV2	TRDJ1	CACDPLRGDTPRYTDKLIF	0.132703
14	TRDV2	TRDJ1	CACDPLLGDTPRDTDKLIF	0.130913
15	TRDV2	TRDJ1	CACDPLLGDTPRYTDKLIC	0.12989
16	TRDV2	TRDJ1	CACDPLLGDTPRYTDKLIV	0.126566
17	TRDV2	TRDJ1	CARDPLLGDTPRYTDKLIF	0.125543
18	TRDV2	TRDJ1	CACDPLLGDTPRYTDKLIL	0.117106
19	TRDV2	TRDJ1	WACDPLLGDTPRYTDKLIF	0.108924
20	TRDV2	TRDJ1	CACDPRLGDTPRYTDKLIF	0.104577
21	TRDV2	TRDJ1	GACDPLLGDTPRYTDKLIF	0.093838
22	TRDV2	TRDJ1	CTCDPLLGDTPRYTDKLIF	0.090258
23	TRDV2	TRDJ1	CACDLLLGDTPRYTDKLIF	0.087446
24	TRDV2	TRDJ1	CAWDPLLGDTPRYTDKLIF	0.086679
25	TRDV2	TRDJ1	CACDPLLGDTSRYTDKLIF	0.081821
26	TRDV2	TRDJ1	YACDPLLGDTPRYTDKLIF	0.077729
27	TRDV2	TRDJ1	CACDSLLGDTPRYTDKLIF	0.074661
28	TRDV2	TRDJ1	CACDPLLLDTPRYTDKLIF	0.072871
29	TRDV2	TRDJ1	CACYPLLGDTPRYTDKLIF	0.07057
30	TRDV2	TRDJ1	CACDPLLGDTPKYTDKLIF	0.066991
31	TRDV2	TRDJ1	CACDPLLGDTPRYIDKLIF	0.066479
32	TRDV2	TRDJ1	CACDPLLGDTLRYTDKLIF	0.065712
33	TRDV2	TRDJ1	CACDPLLGDTPGYTDKLIF	0.065201
34	TRDV2	TRDJ1	CAYDPLLGDTPRYTDKLIF	0.064434
35	TRDV2	TRDJ1	CACDPLLGGTPRYTDKLIF	0.061621
36	TRDV2	TRDJ1	CACGPLLGDTPRYTDKLIF	0.059576
37	TRDV2	TRDJ1	RACDPLLGDTPRYTDKLIF	0.05932
38	TRDV2	TRDJ1	CACDPLLGDTPRYTDKRIF	0.055996
39	TRDV2	TRDJ1	CACDPLLGDTPRYADKLIF	0.052928
40	TRDV2	TRDJ1	CASDPLLGDTPRYTDKLIF	0.052672
41	TRDV2	TRDJ1	CACDPLLGDTPRCTDKLIF	0.051649
42	TRDV2	TRDJ1	CACDPLLGDTPRYTDKFIF	0.051394
43	TRDV2	TRDJ1	CACDPLLGDAPRYTDKLIF	0.048325
44	TRDV2	TRDJ1	CACDPLLGDTPRYTDKLVF	0.043979
45	TRDV2	TRDJ1	CACDPLLGDTPRYTDKLSF	0.038353
46	TRDV2	TRDJ1	CACDPLLGDTPRYTDRLIF	0.034774
47	TRDV2	TRDJ1	CACDPLLGDTPRYTGKLIF	0.031705
48	TRDV2	TRDJ1	CACDPLLGNTPRYTDKLIF	0.021989
49	TRDV2	TRDJ1	CAFDPLLWDTPRYTDKLIF	0.021222
50	TRDV2	TRDJ1	CACDPPLGDTPRYTDKLIF	0.021222

rank	TRDV	TRDJ	CDRJ	%readas
1	TRGV9	TRGJP	CALWEVQELGKKIKVF	87.66021
2	TRGV9	TRGJP	CALWEVQELGKXIKVF	3.23425
3	TRGV9	TRGJP	CALWEVQELXKKIKVF	2.969439
4	TRGV9	TRGJP	YALWEVQELGKKIKVF	0.401824
5	TRGV9	TRGJP	CALLEVQELGKKIKVF	0.299832
6	TRGV9	TRGJP	CALWEVQELGKKINVF	0.288158
7	TRGV9	TRGJP	CALWEVQELDKKIKVF	0.262967
8	TRGV9	TRGJP	CVLWEVQELGKKIKVF	0.23962
9	TRGV9	TRGJP	CALWEVQELGKKIKIF	0.186166
10	TRGV9	TRGJP	CALWEVQELGKRIKVF	0.181865
11	TRGV9	TRGJP	CALWEVQELGKKIKLF	0.180636
12	TRGV9	TRGJP	CALWEVQELGKKIKVL	0.169577
13	TRGV9	TRGJP	CALGEVQELGKKIKVF	0.140085
14	TRGV9	TRGJP	CALCEVQELGKKIKVF	0.132712
15	TRGV9	TRGJP	CALWEGQELGKKIKVF	0.124111
16	TRGV9	TRGJP	CAVWEVQELGKKIKVF	0.121039
17	TRGV9	TRGJP	CAFWEVQELGKKIKVF	0.107522
18	TRGV9	TRGJP	CALWEVQEVGKKIKVF	0.103835
19	TRGV9	TRGJP	CALWEVQEWGKKIKVF	0.101992
20	TRGV9	TRGJP	CALWEVRELGKKIKVF	0.101378
21	TRGV9	TRGJP	CALWEVQELGKKIRVF	0.089089
22	TRGV9	TRGJP	CALWEVQELGKKIEVF	0.086017
23	TRGV9	TRGJP	CALWEVQELGKKSKVF	0.080488
24	TRGV9	TRGJP	CALWEVQELGKEIKVF	0.079259
25	TRGV9	TRGJP	GALWEVQELGKKIKVF	0.076801
26	TRGV9	TRGJP	RALWEVQELGKKIKVF	0.074344
27	TRGV9	TRGJP	CALWGVQELGKKIKVF	0.073729
28	TRGV9	TRGJP	CAWWEVQELGKKIKVF	0.070657
29	TRGV9	TRGJP	CALWEVQELGKKIKGF	0.068814
30	TRGV9	TRGJP	CSLWEVQELGKKIKVF	0.065742
31	TRGV9	TRGJP	CALWEVQELCKKIKVF	0.065127
32	TRGV9	TRGJP	CALWEVQELGKKIKVC	0.064513
33	TRGV9	TRGJP	CALWEVQELGRKIKVF	0.06267
34	TRGV9	TRGJP	CALWEVQGLGKKIKVF	0.061441
35	TRGV9	TRGJP	WALWEVQELGKKIKVF	0.058983
36	TRGV9	TRGJP	CALWEVQELGKKTKVF	0.057754
37	TRGV9	TRGJP	CALWEVQDLGKKIKVF	0.052225
38	TRGV8	TRGJ1	CATWDYYKKLF	0.052225
39	TRGV9	TRGJP	CTLWEVQELGKKIKVF	0.049153
40	TRGV9	TRGJP	CALWEVQELGKKVKVF	0.047924
41	TRGV9	TRGJP	CASWEVQELGKKIKVF	0.043623
42	TRGV9	TRGJP	CALWEVQELGKKIKAF	0.038708
43	TRGV9	TRGJP	CALWEVQELGKKIKVV	0.038093
44	TRGV9	TRGJP	SALWEVQELGKKIKVF	0.031949
45	TRGV9	TRGJP	CALWEVQELGKKIKVS	0.028263
46	TRGV9	TRGJP	FALWEVQELGKKIKVF	0.027034
47	TRGV9	TRGJP	CALWEVQELGEKIKVF	0.021504
48	TRGV5	TRGJ1	CATNYYKKLF	0.020276
49	TRGV9	TRGJP	CALWEVQELGKKITVF	0.019047
50	TRGV9	TRGJP	CALWEVHELGKKIKVF	0.017818

Table S1. Dominant TCR clonotypes identified in iγδT cells

TCR repertoire sequencing of day-34 iγδT cells derived from the 121-3 γδT-iPSC line. The table lists the top 50 TCRδ and TCRγ clonotypes ranked by read frequency. A single dominant TRDV2-TRDJ1 clonotype and a single dominant TRGV9-TRGJP clonotype represented the majority of reads, consistent with preservation of the parental Vγ9Vδ2 T-cell clonotype during differentiation.
