# Supplementary material for: Allogeneic iPSC-derived γδT cells demonstrate antitumor efficacy against patient-derived tissues
Source: Stem Cell Reports. 2026 Jul 23;21(8):103018. doi: 10.1016/j.stemcr.2026.103018 (PMC13476892; doi:10.1016/j.stemcr.2026.103018)
Supplement: Supplementary file 2 [file mmc2.pdf]

STEM CELL REPORTS ISSCR
Article
OPEN ACCESS

Allogeneic iPSC-derived γδT cells demonstrate antitumor efficacy against patient-derived tissues

Ryoko Futai,[1,2] Michiyo Koyanagi-Aoi,[1,3,4] Aito Nakahashi,[1] Daiki Katsura,[1,5] Manabu Horikawa,[1,6] Kazumasa Horie,[1,6] Yoshihiro Kakeji,[6] Yuzo Kodama,[2] and Takashi Aoi[1,3,4,7,8,*]
[1]Division of Stem Cell Medicine, Graduate School of Medicine, Kobe University, Kobe, Hyogo, Japan
[2]Division of Gastroenterology, Department of Internal Medicine, Kobe University Graduate School of Medicine, Kobe University, Kobe, Hyogo, Japan
[3]Division of Advanced Medical Science, Graduate School of Science, Technology and Innovation, Kobe University, Kobe, Hyogo, Japan
[4]Center for Human Resource Development for Regenerative Medicine, Kobe University Hospital, Kobe, Hyogo, Japan
[5]Division of Urology, Graduate School of Medicine, Kobe University, Kobe, Hyogo, Japan
[6]Division of Gastrointestinal Surgery, Department of Surgery, Graduate School of Medicine, Kobe University, Kobe, Hyogo, Japan
[7]Division of Signal Pathways, Biosignal Research Center, Kobe University, Kobe, Japan
[8]Lead contact
*Correspondence: takaaoi@med.kobe-u.ac.jp
https://doi.org/10.1016/j.stemcr.2026.103018

SUMMARY

Various immunotherapies have been developed to treat malignant tumors, and autologous CAR-T cell therapy is clinically used for certain malignancies. However, their efficacy against solid tumors is limited. γδT cells are recognized for their potent tumor cytotoxicity and MHC-unrestricted activity. We successfully induced γδT cells from iPS cells and demonstrated their cytotoxicity against colorectal cancer cell lines *in vitro*. However, cancer cell lines often lack critical tumor characteristics such as heterogeneity and drug sensitivity. In contrast, patient-derived cancer organoids retain many features of the patient's tumor tissues. This study evaluated the cytotoxicity of allogeneic iγδT cells against cell lines and organoids, *in vitro* and *in vivo*. iγδT cells showed 70%–90% cytotoxicity against cell lines and organoids *in vitro*. *In vivo*, local and intravenous administration of iγδT cells suppressed tumor growth by 70%–100%, highlighting their potential as a novel immunotherapy for colorectal cancer.

INTRODUCTION

Various immune cell therapies have been used to treat malignant tumors (Adachi et al., 2018). In recent years, the remarkable success of autologous chimeric antigen receptor (CAR)-T cell therapy in treating leukemia has increased expectations for immune cell therapy in managing malignancies more broadly; however, the proportion of patients with malignancies who can benefit from immune cell therapies remains limited (Adachi et al., 2018). A particularly significant challenge is their ineffectiveness in treating solid tumors (Adachi et al., 2018). Additionally, obstacles such as the time and cost required to initiate autologous treatment impede their widespread adoption (Rosenberg and Restifo, 2015). Consequently, there is growing interest in allogeneic (off-the-shelf) cell therapy.

We focused on γδT cells as a potential therapeutic modality for colorectal cancer (CRC). γδT cells represent approximately 3–5% of peripheral blood lymphocytes and are capable of targeting various types of tumors in an MHC-unrestricted manner with a single type of γδT cell receptor (Bohme and Pilstrom, 1991). Human γδT cells are broadly classified into two major subsets, Vδ1 and Vδ2, based on δ-chain usage (Vantourout and Hayday, 2013). The Vδ1 subset is predominantly tissue-resident and localizes to epithelial tissues, such as the intestinal mucosa. These cells recognize stress-induced ligands, including MHC class I–related molecules (MICA/B) as well as lipid antigens presented by CD1d (Silva−Santos et al., 2015). The tissue-resident nature of Vδ1 T cells, which express cytotoxic molecules, such as Fas ligand (FasL) and TRAIL, may confer an advantage in tumor immunity (Stary et al., 2024). In contrast, Vγ9Vδ2 cells represent the major γδ T cell population in human peripheral blood, and recognize non-peptidic phosphorylated antigens (PAgs), such as isopentenyl pyrophosphate (IPP) and (E)-4-hydroxy-3-methyl-but-2-enyl pyrophosphate (HMBPP), via the BTN3A1/BTN2A1 complex (Yuan et al., 2023). Importantly, Vγ9Vδ2 T cells can be readily isolated from the peripheral blood and expanded *ex vivo* using well-established protocols. Furthermore, their anti-tumor activity has been extensively investigated in clinical settings (Martinez and Moon, 2019). Based on these advantages, particularly their accessibility, expandability, and established clinical relevance, we selected Vγ9Vδ2 T cells for iPSC derivation and the subsequent functional analysis against CRC (Murai et al., 2023).

CRC is the third most common cancer worldwide, accounting for approximately 10% of all cancer cases, and is the second leading cause of cancer-related death (Organization, 2024). Therefore, the development of novel therapies for CRC, a highly heterogeneous cancer, remains a paramount challenge in global healthcare (Izumi et al., 2013), and γδT cells are considered a promising candidate

Published by Elsevier Inc. on behalf of International Society for Stem Cell Research.
This is an open access article under the CC BY license (http://creativecommons.org/licenses/by/4.0/).

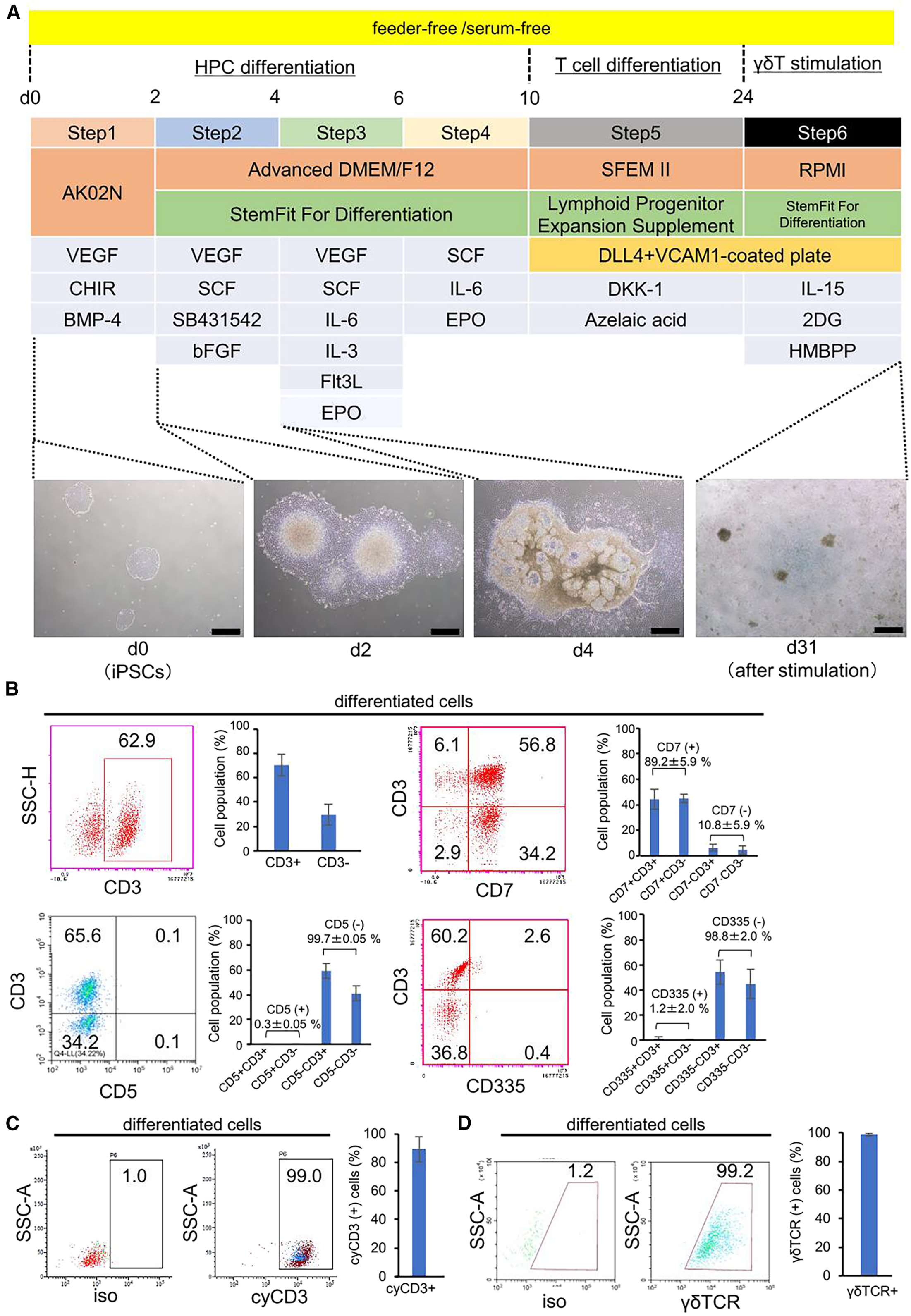

(legend on next page)

modality. Several clinical trials have been conducted using autologous γδT cells derived from peripheral blood and expanded *ex vivo* for treating CRC (Izumi et al., 2013; Nicol et al., 2011). However, autologous transplantation of γδT cells, which constitute less than a few percent of peripheral blood lymphocytes, causes individual differences in the expansion of transplanted γδT cell numbers (Izumi et al., 2013). It also faces challenges such as the depletion of circulating γδT cell numbers and *in vitro* expansion capacity (Nicol et al., 2011). The clinical outcomes associated with these treatments are generally unsatisfactory (Nicol et al., 2011). Although MHC-unrestricted target recognition by γδT cells raises expectations for the efficacy of allogeneic γδT cell therapy and diminishes concerns about graft-versus-host disease (GVHD), peripheral blood-derived γδT cells have limited expansion capabilities, rendering them unsuitable for use as materials in mass-produced allogeneic immune therapeutic modalities.

Our strategy for the mass production of allogeneic γδT cells involves the use of induced pluripotent stem cells (iPSCs), which have unlimited proliferative capacity. We previously reported the generation of Vγ9Vδ2T cell-derived iPSCs and their subsequent differentiation into hematopoietic progenitor cells (Watanabe et al., 2018). In a follow-up study, we successfully regenerated Vγ9Vδ2T cells from these iPSCs (Murai et al., 2023). iPSC-derived γδT cells (iγδTs) exhibit MHC-unrestricted cytotoxicity *in vitro* against various cancer cell lines, including leukemia, hepatic cancer, and colon cancer (Murai et al., 2023). However, a previous study employed differentiation conditions that included xenogeneic serum and feeder cells, and it demonstrated cytotoxicity solely *in vitro*, without extending to *in vivo* evaluations. To address this limitation, and based on prior reports showing that αβ T cells can be generated under xeno-free and feeder-free conditions (Wakao et al., 2013), we hypothesized that γδT cells can be generated from human iPS cells under clinically relevant xeno-free and feeder-free conditions while preserving their intrinsic antitumor functionality.

In this study, we successfully generated iγδTs differentiated from γδT cell-derived iPSCs under feeder-free and xeno-free conditions and systematically evaluated their antitumor activity to test our hypothesis. We demonstrated that these iγδTs exhibit cytotoxic activity against CRC and leukemia cell lines, as well as against patient-derived CRC organoids *in vitro*, while also exerting antitumor effects *in vivo* in xenograft models. Importantly, these cells exhibited no cytotoxicity toward allogeneic normal peripheral blood mononuclear cells (PBMCs). Our findings will pave the way for the realization of off-the-shelf allogeneic γδT cell therapy.

RESULTS

Feeder-free/serum-free differentiation of γδT-iPSCs into iγδT cells

First, we investigated whether γδT-iPSCs can be differentiated into γδT cells under feeder-free and serum-free conditions (Figure 1A). Representative phase-contrast images of iPSCs (day 0) and iPSC derivatives on days 2, 4, and 31 are shown in the lower panels of Figure 1A. Flow cytometry (FCM) showed the differentiated cells after γδT stimulation were approximately 70.4% ± 8.69% positive for CD3, 89.2% ± 5.9% positive for CD7, and almost negative for CD5 (Figure 1B). This expression pattern was similar to that of iγδT cells previously generated using xenogeneic serum on feeder layers (Murai et al., 2023). We further confirmed that iγδT cells could also be induced from two additional iPSC lines by evaluating the $CD3^+$ expression via FCM (Figures S1A and S1B). Using our differentiation protocol, approximately 2.5×10^6 hematopoietic progenitor cells were generated from an initial 1×10^3 iPSCs. From 1.2×10^4 of these progenitors, approximately 8.0×10^5 iγδ T cells were obtained, corresponding to an estimated $\sim 8 \times 10^4$-fold expansion per single iPSC. Although approximately 29.6% ± 8.69% of the differentiated cells were CD3-negative, these cells were negative for CD335 (NKp46), a marker for NK cells, indicating that they were not differentiated into NK cells (Figure 1B). Additionally, an FCM analysis after fixation and membrane permeabilization showed that the rate of positivity for cytoplasmic CD3 (cyCD3) was 89.18% ± 8.5% (Figure 1C). Considering that 98.9% ± 0.59% of the differentiated cells

Figure 1. Feeder-free/serum-free differentiation of γδT-iPSCs into iγδT cells

(A) The protocol for the differentiation of iPSCs into γδT cells. The lower panels display representative phase-contrast images of iPSCs (day 0) and iPSC derivatives (days 2, 4, and 31). Scale bars, 500 μm.

(B) Cell surface markers for pan T cells (CD3, CD7, and CD5) and NK cells (CD335) were analyzed for differentiated cells by flow cytometry on day 39. The graphs on the right side of each FCM show the proportion of CD3-positive and CD3-negative cells (*n* = 4 independent experiments, mean ± SD), as well as the proportion of CD3 and CD7, CD3 and CD5, and CD3 and CD335-positive and-negative cells (*n* = 3 independent experiments, mean ± SD) in the differentiated cells.

(C and D) The expression of cytoplasmic CD3 (cyCD3) (C) and γδTCR (D) was analyzed for differentiated cells by flow cytometry on days 27 and 39, respectively. The left panels show the isotype controls. The graphs on the right show the proportion of cyCD3(+) cells (C) and γδTCR(+) cells (D) in the differentiated cells (*n* = 3 independent experiments; mean ± SD).

Table 1. HLA profiles of iPS cells (62B3), PBMCs from three donors, Jurkat, SW480, and two patient-derived organoids

	HLA-A	HLA-B	HLA-C	HLA-DRB1
PBMC 1	24:02 26:02	37:01 59:01	01:02 06:02	04:05 10:01
PBMC 2	02:01 24:02	54:01 -	01:02 12:02	04:05 15:01
PBMC 3	24:02 31:01	07:02 51:01	07:02 14:02	01:01 11:01
Jurkat	03:01 -	07:02 35:03	04:01 07:02	07:01 15:01
SW480	02:01 24:02	07:02 15:18	07:02 07:04	01:03 13:01
62B3	02:01 24:02	40:01 54:01	01:02 03:04	04:03 04:05
Rm8	24:02 -	59:01 -	01:02 -	04:05 -
Rm19	02:07 24:02	46:01 52:01	01:02 12:02	15:02 16:02

HLA profiles of iPS cells (62B3) before iγδT cell differentiation induction, peripheral blood-derived PBMCs (collected from three individuals), Jurkat, SW480, and patient-derived organoids (two cases).

were γδTCR-positive (Figure 1D), we successfully generated iγδT cells under feeder-free and serum-free conditions. Regarding CD3-negative cells, more than 90% were CD7-positive and 99% were positive for cyCD3, indicating that these cells retain the T-lineage characteristics despite lacking surface CD3 expression.

We further characterized the phenotypes of the iγδT cells generated in this study. γδTCR expression was markedly higher than that in peripheral blood-derived γδT cells (PBγδT cells), as demonstrated by a substantially higher mean fluorescence intensity (MFI) and a single, uniform peak, in contrast to the bimodal distribution observed in peripheral γδT cells (Figure S1C). In addition, a TCR repertoire analysis showed the exclusive expression of TRGV9 and TRDV2, indicating a highly restricted Vγ9Vδ2 TCR repertoire consistent with a single dominant clone (Figure S1D; Table S1). The CD4/CD8 expression dynamics were evaluated at both the differentiation stage (day 26) and final product stage (day 58) following separation into $CD3^+$ and $CD3^-$ fractions. At both stages, the CD4 expression was minimal, whereas CD8α was expressed in approximately 30%–40% of cells, with negligible CD8β expression, indicating a consistent $CD4^-CD8\alpha^+CD8\beta^-$ phenotype, irrespective of the surface CD3 expression (Figure S1E). Furthermore, more than 90% of iγδT cells expressed NK cell-activating receptors, including NKG2D, DNAM-1, and NKp30 (Figure S1F). The majority of iγδT cells displayed a terminal effector phenotype characterized by the $CD45RA^+/CD27^-$ expression (Odaira et al., 2016) (Figure S1G) and produced IFN-γ as well as cytotoxic molecules such as perforin and granzyme B upon stimulation by co-culturing with Jurkat cells in the presence of brefeldin A (Figure S1H).

iγδT cells exhibit cytotoxicity against allogenic cancer cell lines *in vitro*

To evaluate the cytotoxicity of iγδT cells induced under xeno-free conditions against cancer cell lines, we labeled Jurkat and SW480 cells with Venus-Akaluc, which allows live cells to be monitored by fluorescence (Venus) or bioluminescence signal after AkaLumine or AkaLumine-HCl addition (Akaluc). Hereafter, cells transfected with Venus-Akaluc are referred to as "JurkatA" and "SW480A" cells. First, we divided iγδT cells into cell surface CD3-positive and CD3-negative cells (Figure S2A) as effectors (E) and co-cultured them with JurkatA cells as target cells (T) at E:T ratios of 4:1, 2:1, and 1:1. The luminescence intensity of the "no effector" group, which was not treated with iγδT cells, was set to 100%, and the relative luminescence intensity was measured (Figure S2B). At all E:T ratios, the cytotoxicity of the cells on CD3-positive cells was significant ($p < 0.05$). Moreover, CD3-negative cells also exhibited significant cytotoxicity ($p < 0.05$) at E:T ratios of 4:1 and 2:1. From these results, we found that both cell surface CD3-positive and -negative cells had cytotoxic activity, although there were differences in the strength of the cytotoxicity. The following experiments were performed using unsorted iγδT cells.

The cytotoxicity of iγδT cells was assessed by co-culturing them with JurkatA cells. We confirmed that the HLA of Jurkat and iγδT cells did not match (Table 1). The fluorescence of JurkatA cells (T) decreased in a dose-dependent manner with increasing concentrations of iγδT cells (E) (Figure 2A). After 5 days of co-culture, iγδT cells exhibited significant cytotoxicity against JurkatA cells with an average killing rate of 97% and 90% at E:T ratios of 4:1 and 2:1, respectively (Figure 2B). In addition, treatment with zoledronic acid did not result in a statistically significant difference in cytotoxicity (data not shown).

Next, the cytotoxicity of iγδT cells toward solid tumor cells was evaluated by co-culturing them with the colon cancer cell line (SW480A) using the xCELLigence system. We confirmed that the HLA of SW480A cells and iγδT cells did not match (Table 1). In the cytotoxicity assay using xCELLigence, iγδT cells exhibited significant cytotoxicity against SW480A, with killing rates of 93.7% ± 9.9% and 77.7% ± 10.4% at E:T ratios of 2:1 and 1:1, respectively (Figures 2C and 2D). To compare the cytotoxicity of iγδT cells and peripheral blood-derived γδT cells (PBγδT cells), the cells were co-cultured with SW480A. PBγδT cells were generated by stimulating PBMCs with HMBPP and IL-2 for 7 days. In the xCELLigence assay, iγδT cells exhibited 82.8% ± 15.7% cytotoxicity at an E:T ratio of 2:1, while PBγδT cells exhibited 53.7% ± 14.6%. At an E:T ratio of 1:1, iγδT cells exhibited 59.7% ± 23.7% cytotoxicity, whereas PBγδT cells exhibited 28.3% ± 30.0% (Figure 2E). No significant difference in cytotoxicity was observed

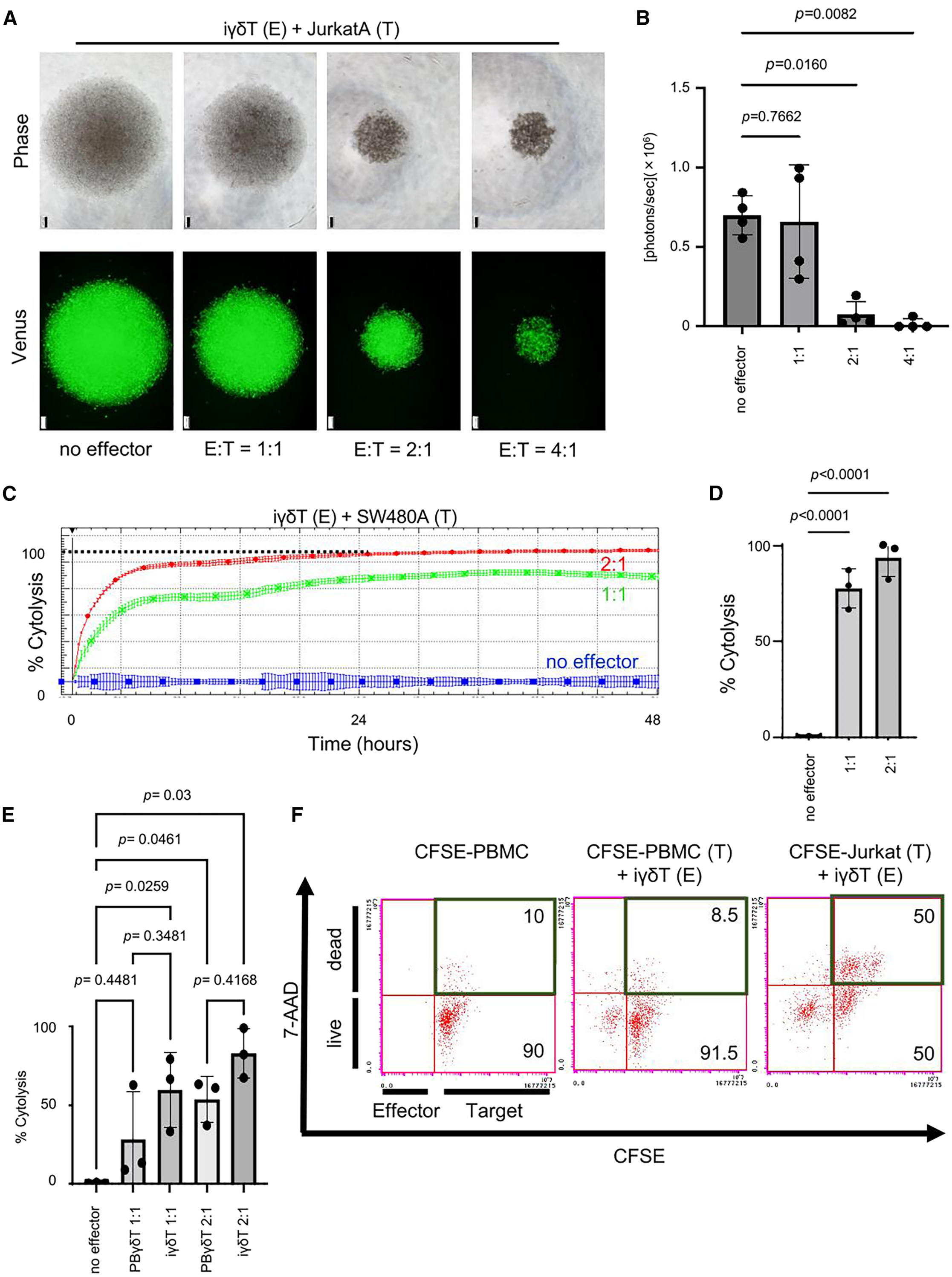

Figure 2. iγδT cells demonstrate cytotoxicity against cancer cell lines but not against normal PBMCs

(A) Representative phase-contrast (upper panels) and fluorescence microscopy (lower panels) images of *in vitro* co-culture of JurkatA cells and iγδT cells. Scale bars, 200 μm.

(B) The AkaBLI assay for co-culture of JurkatA and iγδT cells. Luminescence intensity on day 5 was quantified. Significant differences were observed at an E:T ratio of 4:1 ($p = 0.0082$) and 2:1 ($p = 0.0160$).

(legend continued on next page)

between iγδT and PBγδT cells at E:T ratios of 2:1 and 1:1. These results indicate that the cytotoxicity of iγδT cells was not inferior to that of PBγδT cells (Figure 2E).

Next, to evaluate the cytotoxicity of iγδT cells against normal cells, a mixed lymphocyte reaction (MLR) assay was performed using allo PBMC as the target and iγδT cells as the effector. The HLA of PBMC and iγδT cells did not match each other (Table 1). After iγδT cells were co-cultured with Carboxy fluorescein diacetate succinimidyl ester (CFSE)-labeled Jurkat for one day, the proportion of 7-AAD+ dead cells was 50% (Figure 2F, right panel). On the other hand, iγδT cells were co-cultured with CFSE-labeled PBMCs from a healthy donor, and the proportion of 7-AAD+ dead cells among the target PBMC (CFSE positive cells) was 8.5% (Figure 2F, middle panel). This cell death rate was almost the same as when PBMCs were cultured alone (10%, Figure 2F, left panel). We also performed MLR assays using PBMCs from two additional independent donors. The iγδT cells consistently exhibited a low alloreactivity toward all donors tested, with proliferative responses significantly lower than those observed in response to Jurkat cells (Figure S2C). These results indicate that iγδT cells do not attack normal PBMCs, even those with different HLAs.

iγδT cells exhibit cytotoxicity against allo cancer cell lines *in vivo*

Next, we evaluated *in vivo* cytotoxicity of iγδT cells against cancer cell lines in immunodeficient mice. iγδT cells (E) and JurkatA cells (T) were co-administered subcutaneously to immunodeficient mice at an E:T ratio of 2:1, and we monitored the tumor burden in mice weekly for 5 weeks using the AkaBLI system (Figure 3A). Because iγδT cells are generated through a multi-step differentiation process from hiPSCs, potential variability between independent differentiation experiments represents an important consideration when evaluating their antitumor activity. To first assess the magnitude of the antitumor effect and the degree of inter-animal variability, we performed a pilot experiment using iγδ T cells derived from a single differentiation, in which three mice were treated and compared with three control mice (Figure S3A). As shown in Figure S3B, iγδ T cells exhibited a clear and statistically significant tumor-suppressive effect with limited inter-animal variability. Based on these results, subsequent experiments were designed to evaluate reproducibility across independent differentiations rather than relying solely on technical replication within a single differentiation. Specifically, the iγδ T cells derived from three or more independent differentiation experiments were tested, with each differentiation evaluated in one treated mouse and one control mouse. iγδT cells induced in three independent experiments were used, and mice transplanted with JurkatA cells alone served as controls for each experiment. Immunostaining of the extracted tumor tissue with or without iγδT cells revealed that both were CD3 positive (Figure S4A). This indicated the presence of JurkatA cells. When iγδT cells were co-administered, tumor growth was suppressed (Figure 3B), and across the three independent datasets ($n = 1$ per group), the tumor weights in the mice treated with iγδT cells were 83%, 97%, and 91% lower, respectively, than those in the corresponding control mice (Figure 3C). In the iγδT cell-treated group, the bioluminescence intensity was lower than that in the untreated group (Figure 3D). Notably, a tumor that showed luminescence three weeks after injection was observed to shrink again (Figure 3A), suggesting that iγδT cells functioned *in vivo* for at least three weeks. After five weeks, the luminescence intensities in the iγδT cell-treated mice were 100%, 98%, and 83% lower, respectively, than those in the corresponding control mice. (Figure 3E). Immunostaining of tumors using a TCR gamma/delta monoclonal antibody in the iγδT cell-treated group showed the presence of T cells around the tumor (Figure 3F).

Next, iγδT cells (E) and SW480A (T), as solid tumor cell lines, were co-administered subcutaneously to immunodeficient mice at an E:T ratio of 5:1, and the mice were observed weekly for four weeks (Figure 3G). Immunostaining of tumor tissue revealed panCK positivity (Figure S4B) in the presence or absence of iγδT cells. This indicated the presence of SW480A cells. When iγδT cells were co-administered, tumor growth was suppressed (Figure 3H), and across the three independent datasets, the tumor weights in the iγδT cell-treated mice were 80%, 84%, and 91% lower, respectively, than those in the corresponding control mice (Figure 3I). The bioluminescence intensity was lower in the iγδT cell-treated group than that in the untreated group (Figure 3J). After four weeks, the luminescence intensities in the iγδT cell-treated mice were 76%, 100%, and 100% lower, respectively, than those in the corresponding control mice (Figure 3K). CD3 immunostaining of tumors in the iγδT cell-treated group showed the presence of T cells around the tumor (Figure 3L).

(C) The cytotoxicity of iγδT cells against SW480A cells was assessed using xCELLigence.

(D) The cytotoxicity after two days of co-culture of iγδT cells and SW480A cells was quantified and compared. Significant differences were observed at an E:T ratio of 2:1 ($p < 0.0001$) and 1:1 ($p < 0.0001$).

(E) The cytotoxicity of iγδT cells and peripheral blood-derived γδT cells (PBγδT cells) against SW480A was compared using xCELLigence.

(F) A representative example compares the cytotoxicity of iγδT cells against CFSE-labeled PBMCs and CFSE-labeled Jurkat cells using FCM.

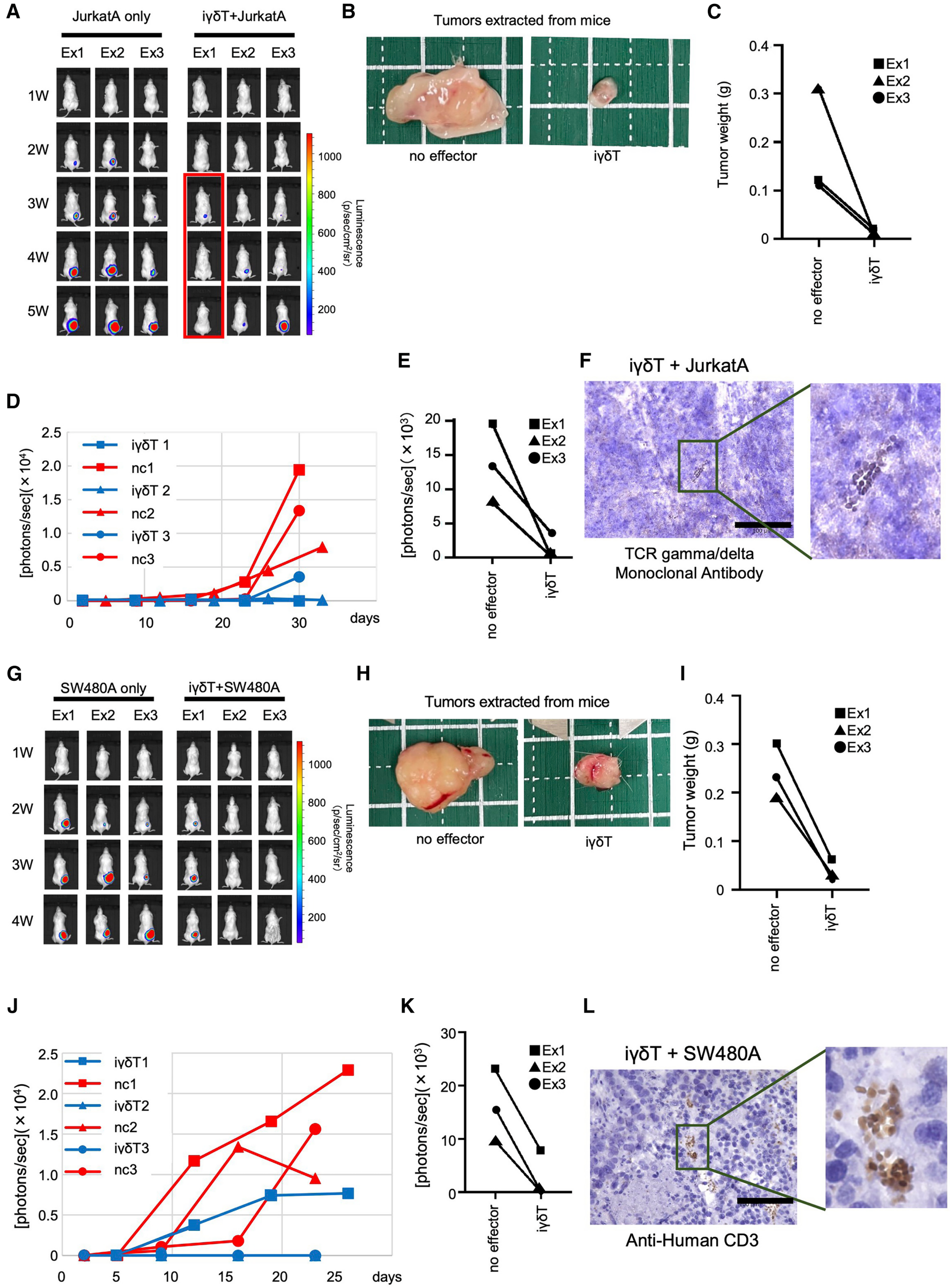

Figure 3. iγδT cells also demonstrate cytotoxicity against cancer cell lines *in vivo*

(A) Weekly time-lapse images of bioluminescence signals of injected JurkatA cells with or without iγδT cells (n = 3).

(B) Representative image of tumors with no effector (left panel) and with iγδT cells (right panel) excised from mice.

(C) The tumor weights measured 30–32 days after the subcutaneous transplantation of JurkatA cells with or without iγδT cells.

(legend continued on next page)

iγδT cells exhibit cytotoxicity against patient-derived colorectal cancer organoids

A significant limitation of cancer cell lines is that they lack many characteristics of actual clinical tumors and their sensitivity to anticancer agents (Toshimitsu et al., 2022). Therefore, we evaluated the cytotoxicity of iγδT cells against patient-derived organoids that retain more characteristics of patient tumor tissues than cancer cell lines.

First, we examined the cytotoxicity of iγδT cells in two patient-derived CRC organoids (Rm8 and Rm19) *in vitro*. The HLA of the patient-derived organoids (two cases) is shown in Table 1. The HLA of iγδT cells does not match that of Rm8 or Rm19. As with its introduction into cancer cell lines, Venus-Akaluc was introduced into patient-derived CRC organoids, hereafter referred to as Rm8A and Rm19A.

Rm8A and Rm19A were co-cultured with iγδT cells *in vitro* at an E:T ratio of 10:1. Representative images of Rm8A and Rm19A after four days of co-culture are shown (Figures 4A and 4B, respectively). An ATP assay conducted after four days of co-culture revealed that Akaluc luminescence decreased by an average of 74% for Rm8A (Figure 4C) and by an average of 94% for Rm19 A (Figure 4D). For Rm8A, the difference was less visible in the Venus fluorescence images (Figure 4A); however, a quantitative analysis of Akaluc luminescence showed a significant difference in luminescence intensity (Figure 4C). These results indicate that iγδT cells exerted significant cytotoxicity against both patient-derived CRC organoids *in vitro*.

Next, we evaluated the cytotoxicity of iγδT cells in patient-derived CRC organoids *in vivo*. Rm8A and iγδT cells were co-injected subcutaneously into immunodeficient mice at an E:T ratio of 10:1. Immunostaining of the resulting tumor tissue revealed the presence of panCK-positive cells (Figure S5A). This indicated the presence of Rm8A organoids. Three sets of weekly images captured using IVIS are shown (Figure 4E). In the iγδT cell-treated group, the bioluminescence intensity of the tumor was lower than that in the untreated group (Figure 4F). At six weeks, the luminescence intensities in the iγδT cell-treated mice were 100%, 95%, and 94% lower, respectively, than those in the corresponding control mice across the three independent datasets. (Figure 4G). Immunostaining of the tumor with CD3 in the iγδT cell-treated group revealed the presence of CD3-positive T cells surrounding the tumor (Figure 4H).

Similarly, Rm19 A and iγδT cells were injected subcutaneously into immunodeficient mice at an E:T ratio of 10:1. Immunostaining of the resulting tumor tissue revealed panCK-positive cells (Figure S5B). This indicated the presence of Rm19A organoids. Three sets of weekly images captured using the IVIS are shown (Figure 4I). In the iγδT cell-treated group, the bioluminescence intensity of the tumor was lower than that in the untreated group (Figure 4J). At six weeks, the luminescence signals in the iγδT cell-treated mice were below the detection limit in all three independent datasets, whereas clear signals were detected in the corresponding control mice across the three independent datasets. (Figure 4K).

Immunostaining of the tumor with CD3 in the iγδT cell-treated group revealed CD3-positive T cells surrounding the tumor (Figure 4L).

Intravenously administered iγδT cells exhibit cytotoxicity against patient-derived organoid Rm8A

When considering clinical settings, the use of iγδT cells is expected primarily for cases with metastasis or dissemination; therefore, systemic administration rather than just local control is desirable. Therefore, we evaluated the cytotoxicity of intravenously administered iγδT cells via retro-orbital injection. A patient-derived CRC organoid, Rm8A, was transplanted subcutaneously into immunodeficient mice, and iγδT cells were simultaneously injected intravenously at an E:T ratio of 10:1. The intravenous injection was repeated once a week, and the mice were observed for four weeks. Figure 5A shows a schematic representation of the concurrent intravenous injection of iγδT cells into mice subcutaneously transplanted with Rm8A. Four weekly IVIS images are shown (Figure 5B). Representative

(D) Quantification of temporal changes in bioluminescence intensity from JurkatA tumors in mice. "nc" stands for "no effector control".
(E) The bioluminescence intensity in JurkatA-transplanted mice immediately before euthanasia.
(F) A representative immunohistochemical staining image of JurkatA tumors excised from mice on day 30. A TCR gamma/delta monoclonal antibody was used. Scale bars, 100 μm.
(G) Weekly time-lapse images of bioluminescence signals of injected SW480A cells with or without iγδT cells (n = 3).
(H) A representative image of SW480A tumors with no effectors (left panel) and with iγδT cells (right panel) excised from mice.
(I) The tumor weights collected 23–26 days after the subcutaneous transplantation of SW480A cells with or without the co-administration of iγδT cells.
(J) Quantification of temporal changes in bioluminescence intensity from SW480A tumors in mice. "nc" stands for "no effector control".
(K) The bioluminescence intensity in the SW480A-transplanted mice was measured immediately before euthanasia.
(L) A representative immunohistochemical staining image of SW480A tumors excised from mice on day 26. Anti-Human CD3 antibody was used. Scale bars, 100 μm. In panels C, E, I, and K, each pair of connected data points represents a dataset consisting of one mouse treated with the iγδT cells derived from a single independent differentiation experiment and one corresponding untreated control mouse.

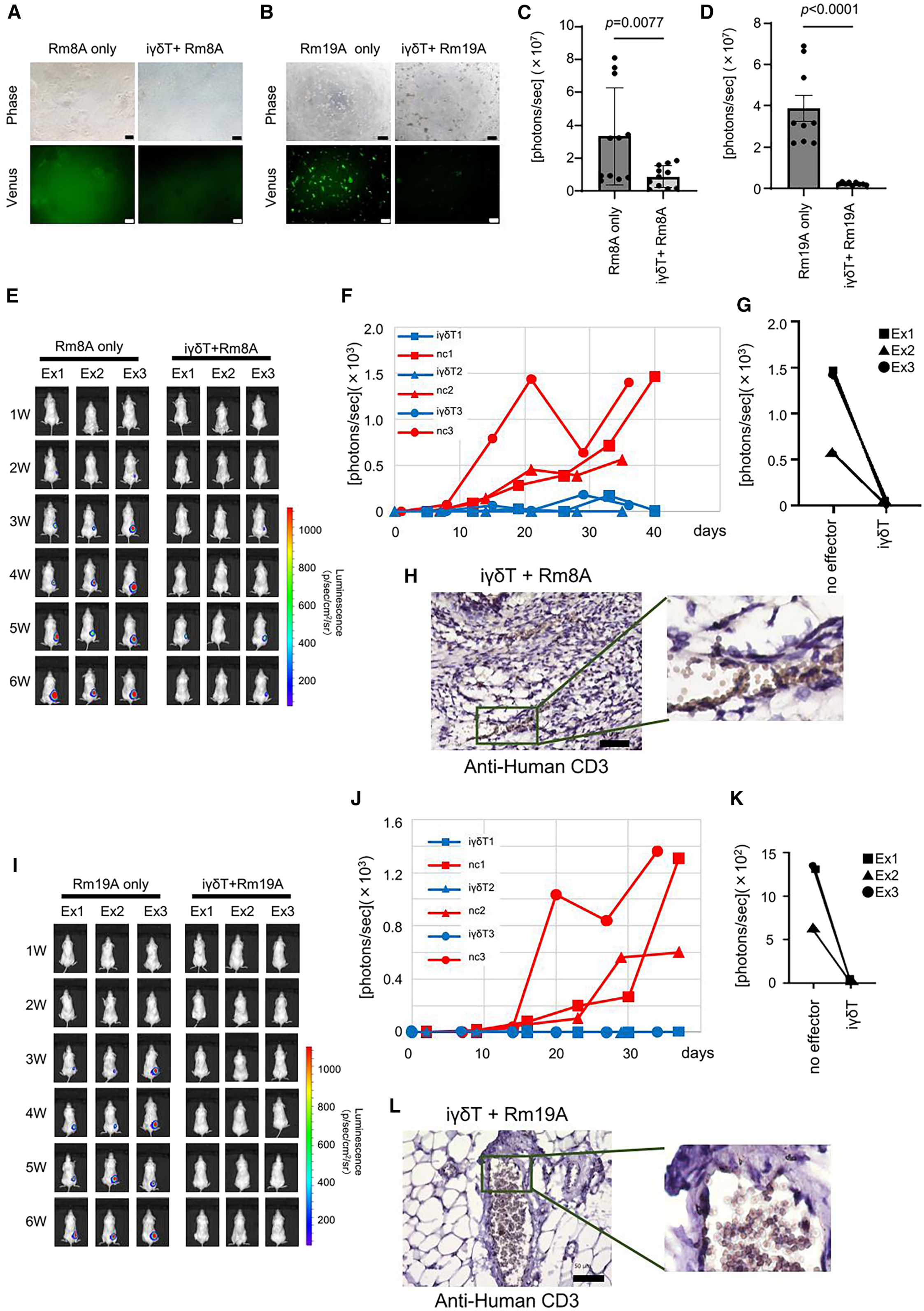

(legend on next page)

images of the extracted tumors, with or without iγδTs, are shown in Figure 5C. Immunostaining of tumor tissue revealed panCK-positive cells (Figure S6A). This indicated the presence of Rm8A organoids. In the iγδT cell-injected group, tumor growth was suppressed (Figure 5C), and across the four independent datasets, the tumor weights in the iγδT cell-treated mice were 40%, 52%, 25%, and 88% lower, respectively, than those in the corresponding control mice (Figure 5D). Bioluminescence intensity was lower in the iγδT cell-treated group than in the untreated group (Figure 5E). After four weeks, the Akaluc luminescence intensities in the iγδT cell-treated mice were 97%, 53%, 71%, and 89% lower, respectively, than those in the corresponding control mice. (Figure 5F). Immunostaining for CD3 in the tumor extracted from the iγδT cell-treated group revealed the presence of T cells surrounding the tumor (Figure 5G).

Next, iγδT cells were administered after confirming tumor engraftment. Simultaneous intravenous administration was designed to model adjuvant therapy following R0 resection, in which no macroscopic tumor is detectable, whereas post-engraftment treatment models the therapy for advanced or recurrent tumors. The patient-derived CRC organoid Rm8A was transplanted subcutaneously into immunodeficient mice, and tumor engraftment was confirmed via IVIS imaging before the intravenous administration of iγδT cells. The exact E:T ratio at the time of administration was unclear, but iγδT cells at 40 times the number of cells in the Rm8A transplant were administered weekly for a total of three doses. Figure 5H shows a schematic representation of the intravenous administration of iγδT cells following the confirmation of tumor engraftment in mice subcutaneously transplanted with Rm8A. Four weekly IVIS images are shown (Figure 5I). Representative images of the extracted tumors with or without iγδTs are shown in Figure 5J. Immunostaining of tumor tissue revealed panCK-positive cells (Figure S6B). This indicated the presence of Rm8A organoids. In the iγδT cell-treated mice, tumor growth was suppressed (Figure 5J), and the tumor weights were 82%, 92%, and 43% lower, respectively, than those in the corresponding control mice across the three independent datasets (Figure 5K). The bioluminescence intensity in the iγδT cell-treated group was lower than that in the untreated group (Figure 5L). At four weeks, the luminescence intensities in the iγδT cell-treated mice were 76%, 78%, and 67% lower, respectively, than those in the corresponding control mice. (Figure 5M). Immunostaining for CD3 in the tumor extracted from the iγδT cell-treated group indicated the presence of T cells surrounding the tumor (Figure 5N).

DISCUSSION

In this study, we successfully induced efficient differentiation of iPSCs into γδT cells under feeder-free and serum-free conditions. Previous reports have documented T cell differentiation from ESCs (Timmermans et al., 2009; Kennedy et al., 2012) and iPSCs (Wakao et al., 2013; Nishimura et al., 2013; Vizcardo et al., 2013; Themeli et al., 2013; Maeda et al., 2016; Minagawa et al., 2018) using feeder cells and without feeder cells (Iriguchi et al., 2021; Trotman−Grant et al., 2021), (Michaels et al., 2022). To the best of our knowledge, this is the first study to report the successful induction of differentiation of γδT cells from iPS cells under feeder-free, serum-free conditions. Currently, the cell-surface CD3 positivity of our differentiated cells is approximately 70.4% ± 8.69%, but nearly all cells showed intracellular CD3 positivity. Notably, more than 90% of the cell-surface CD3-negative cells were CD7-positive, supporting their identity as cells within the T cell lineage, despite a reduced or absent surface CD3 expression. These cells exhibited cytotoxic activity comparable to that of CD3-positive cells (Figure S2B). The mechanisms underlying this heterogeneity in the surface CD3 expression remain to be elucidated, but they may

Figure 4. Locally administered iγδT cells demonstrate cytotoxicity against patient-derived colorectal cancer organoids both *in vitro* and *in vivo*

(A and B) Phase-contrast (upper) and fluorescence (lower) microscopy images of Rm8A (A) and Rm19A (B) colorectal cancer organoids with or without iγδT cells (right and left panels, respectively) *in vitro*. Scale bars, 100 μm (A), 200 μm (B).

(C and D) The bioluminescence intensity of Rm8A (C) and Rm19A (D) colorectal cancer organoids was measured after co-culture with or without iγδT cells for 5 days using an ATP assay. Data are presented as the mean ± SD, with a sample size of $n = 11$ for (C) and $n = 9$ for (D).

(E and I) Weekly time-lapse images of bioluminescence signals of Rm8A (E) and Rm19A (I) colorectal cancer organoids transplanted with or without iγδT cells locally at the time of transplantation ($n = 3$).

(F and J) Temporal changes of bioluminescence intensity from Rm8A (F) and Rm19A (J) colorectal cancer organoids-transplanted mice. "nc" stands for "no effector control".

(G and K) The bioluminescence intensity was measured 6 weeks after the transplantation of Rm8A (G) and Rm19A (K) colorectal cancer organoids. Each pair of connected data points represents a dataset consisting of one mouse treated with the iγδT cells derived from a single independent differentiation experiment and one corresponding untreated control mouse.

(H and L) A representative immunohistochemical staining images of Rm8A (H) and Rm19A (L)-derived tumors excised from mice on day 35 (H) and on day 37 (L) treated with or without iγδT cells. The Anti-Human CD3 antibody was used. Scale bars, 50 μm.

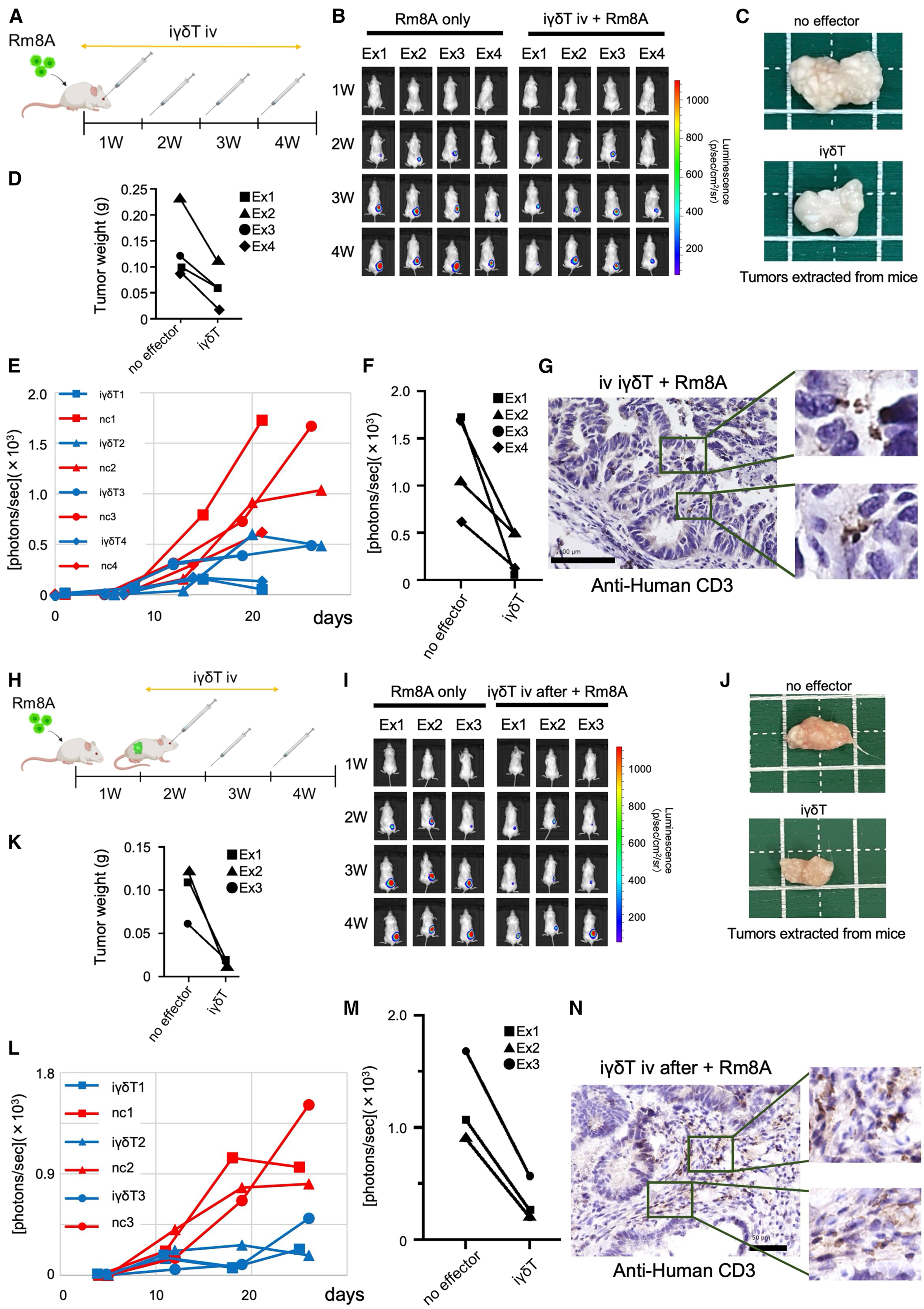

(legend on next page)

include activation-dependent internalization and the downmodulation of CD3(Park et al., 2023).

We utilized iγδT cells as an immune therapeutic approach for CRC, demonstrating their cytotoxicity against both cancer cell lines and patient-derived CRC organoids, *in vitro* and *in vivo*. Previous studies, such as those on hiPSC-derived CAR-T cells, have shown therapeutic efficacy in tumor-bearing animal models transplanted with cancer cell lines from liver cancer (HepG2 and JHH7) and ovarian cancer (KOC7c) (Ueda et al., 2023). Cancer cell lines often lack many clinical tumor characteristics and drug sensitivities (Toshimitsu et al., 2022), whereas patient-derived organoids and PDXs more accurately reflect the properties of actual cancers (Toshimitsu et al., 2022). To our knowledge, no studies have evaluated the cytotoxicity of γδT cells, αβT cells, or CAR-T cells derived from iPS cells against patient-derived organoids, xenografts of CRC, or any other cancer. Our current findings provide robust preclinical evidence supporting the efficacy of T cell therapy for CRC.

In the present study, we used only two patient-derived CRC organoids. Although iγδT cells demonstrated high efficacy in these instances, the number of cases was inadequate. Cancer, even when originating from the same organ, often shows varied genetic and biological traits across patients (Toshimitsu et al., 2022). CRC exhibits high patient prevalence and is characterized by heterogeneity and molecular complexity, resulting in highly variable responses to drug therapy (Saoudi Gonzalez et al., 2023). Individualized evaluation of therapeutic effects using patient-derived specimens could lead to more efficient treatment. By increasing the number of patient-derived organoid cases and elucidating the mechanisms underlying differences in therapeutic outcomes, these organoids could potentially serve as companion diagnostics, and such an approach may enable targeted interventions against factors that diminish treatment efficacy.

Although our current study focused on CRC, a previous report (Murai et al., 2023) and our ongoing analyses suggest that iγδ T cells may exhibit cytotoxic activity against a broader range of tumor types. We are currently extending these investigations to additional cancers, including lung and bladder cancers, and will further explore tumor type specificity in future studies. In addition, to expand the therapeutic indications of iγδT cells, in our blocking experiments, the addition of a neutralizing anti-FASL antibody resulted in a reduction in the cytotoxic activity, whereas blockade of γδTCR or NKG2D did not lead to a noticeable decrease in cytotoxicity (data not shown). These findings suggest that FAS/FASL-mediated interactions may contribute, at least in part, to iγδT cell-mediated cytotoxicity, while the contribution of γδTCR and NKG2D under the present conditions appears limited. Further investigation is required to elucidate the detailed mechanisms mediating iγδT cell-induced cytotoxicity.

γδ T cells are often discussed in terms of "innate-like" versus "adaptive-like" properties. However, these terms do not represent strictly defined developmental lineages, particularly in human γδ T cell biology. Rather, they describe a functional spectrum reflecting differences in TCR repertoire diversity, effector programming, and dependence on antigen-driven clonal expansion (Holtmeier and Kabelitz, 2005; Papadopoulou et al., 2020). In this context, the iγδT cells generated in the present study exhibit

Figure 5. Intravenously administered iγδT cells also demonstrate cytotoxicity against patient-derived colorectal cancer organoids
(A) A schematic diagram of intravenous administration of iγδT cells simultaneously with Rm8A transplantation into mice.
(B) Weekly time-lapse images of bioluminescence signals in immunodeficient mice transplanted with Rm8A, with the simultaneous intravenous administration of iγδT cells (n = 4).
(C) A representative image of tumors with no effector (upper panel) and with iγδT cells (lower panel) excised from mice.
(D) The tumor weights of Rm8A colorectal cancer organoids at the experimental endpoint.
(E) Temporal changes of Akaluc luminescence intensity in Rm8A-transplanted mice. "nc" stands for "no effector control".
(F) The Akaluc bioluminescence intensity was measured 4 weeks after the transplantation of Rm8A colorectal cancer organoids.
(G) A representative immunohistochemical staining image of Rm8A tumors excised from mice on day 24 treated with iγδT cells. The anti-human CD3 antibody was used. Scale bars, 100 μm.
(H) A schematic diagram of intravenous administration of iγδT cells after confirming engraftment after Rm8A transplantation into mice.
(I) Weekly time-lapse images of Akaluc luminescence in immunodeficient mice transplanted with Rm8A, with iγδT cells administered intravenously after the confirmation of tumor engraftment (n = 3).
(J) A representative image of tumors with no effector (upper panel) and with iγδT cells (lower panel) excised from mice.
(K) The tumor weights of Rm8A the colorectal cancer organoids at the experimental endpoint.
(L) Temporal changes of Akaluc luminescence intensity in Rm8A-transplanted mice. "nc" stands for "no effector control".
(M) The Akaluc bioluminescence intensity was measured 4 weeks after the transplantation of Rm8A colorectal cancer organoids.
(N) A representative immunohistochemical staining image of Rm8A tumors excised from mice on day 24 treated with iγδT cells. The Anti-Human CD3 antibody was used. Scale bars, 50 μm. In panels D, F, K, and M, each pair of connected data points represents a dataset consisting of one mouse treated with the iγδT cells derived from a single independent differentiation experiment and one corresponding untreated control mouse.

multiple features characteristic of an innate-like γδ T cell program. iγδT cells uniformly express the Vγ9Vδ2 TCR, a subset known for semi-invariant TCR usage and rapid, largely antigen-independent effector responses (Davey et al., 2017; Papadopoulou et al., 2020). In addition, iγδT cells include a CD8αα$^+$ population while lacking CD8β expression, and they express multiple NK receptors, consistent with innate-like effector differentiation. Furthermore, functional phenotyping based on the CD27 and CD45RA expression reveals that the vast majority of iγδT cells adopt a terminal effector (TEMRA) phenotype, indicating the acquisition of a pre-armed effector function rather than progressive antigen-driven memory diversification. Together, these observations suggest that iγδT cells are developmentally and functionally biased toward an innate-like γδ T cell program, thus providing a mechanistic basis for their rapid effector capacity. This innate-like functional bias may underlie the rapid and robust cytotoxic responses observed in our CRC models.

The iγδT cells developed in this study exhibited significant efficacy against CRC without the need for "armoring" strategies such as forced cytokine expression, immune checkpoint inhibition, or transduction of CAR or TCR targeting specific cancer antigens. A primary challenge in T cell therapy is maintaining the viability and function of the administered T cells over the long term. T cells subjected to prolonged and chronic antigen stimulation enter a state of "exhaustion," characterized by changes in transcription and epigenetics (Blank et al., 2019; Wherry and Kurachi, 2015; Wherry et al., 2007; Sen et al., 2016). This state leads to reduced proliferative capacity and effector functions through multiple mechanisms, including diminished cytokine secretion (Yu et al., 2023), metabolic unfitness (Zhao et al., 2024), and upregulation of immunosuppressive receptors. Although CAR T cell therapy has achieved remarkable clinical success in treating B-cell malignancies, its efficacy against solid tumors remains limited (Zhao et al., 2024). A study has reported that CAR-T cells without armoring could suppress tumor growth for only approximately 10 days, failing to achieve sustained tumor regression (Zhao et al., 2024). Although antibodies targeting PD-1, a key molecule in inhibitory signaling, have shown efficacy in some cases, fully exhausted T-cells do not restore the function with PD-1 antibody alone (Kristen E. Pauken et al., 2016). Considerable advancements have been made in CAR-T cell technology to produce cells with enhanced persistence/expansion and antitumor responses (Alizadeh et al., 2019). Reports have suggested that preconditioning T cells with IL-15, overexpressing IL-15 or IL-10, or deleting ZC3H12A (also known as REGNASE-1), which degrades the mRNA of immune-activating cytokines, could enhance T cell antitumor immunity (Zhao et al., 2024; Alizadeh et al., 2019; Hurton et al., 2016; Wei et al., 2019). Addressing T cell exhaustion to further augment its efficacy presents a future challenge.

The iγδT cells generated in this study did not exhibit cytotoxicity against allogeneic PBMCs, which are normal cells of different HLA types. Currently available treatments, including autologous CAR T cell therapy, TCR T cell therapy, and CAR NK-cell therapy, necessitate genetic engineering of a patient's own T cells (Themeli et al., 2013), leading to high costs and treatment delays. Approaches that introduce CAR genes or TCRs into allogeneic iPSC-derived T cells have been explored (Yale S. Michaels et al., 2022); however, these approaches require TCR knockout to mitigate the risk of GVHD (Iriguchi et al., 2021). In recent years, NK and γδT cells have been considered as alternative sources for CAR-based cell therapy. Unlike αβT cells, neither NK cells nor γδT cells can cause GVHD (Bohme and Pilstrom, 1991; Zhang et al., 2023; Tsiverioti et al., 2024). They also exhibit cancer-killing activity via CAR-independent innate mechanisms, in addition to CAR-dependent target recognition. The persistence of NK cells in the absence of cytokine support is limited, and their ability to infiltrate tumors is low (Zhang et al., 2023; Tsiverioti et al., 2024). Conversely, γδT cells can infiltrate tumors (Zhang et al., 2023), participate in antibody-dependent cellular cytotoxicity (ADCC), cross-present antigens, and orchestrate interactions with other immune cells in the TME (Tsiverioti et al., 2024). Our iγδT cells could potentially serve as carriers for CAR gene introduction, and we have initiated a project on CAR-expressing iγδT cells (data not shown). To date, no CAR T cell therapy has been approved for CRC. Although candidates targeting CEA and GUCY2C are under investigation, their efficacy in solid tumors remains limited owing to antigen escape and the immunosuppressive tumor microenvironment (Martinez and Moon, 2019). The development of allogeneic CAR-iγδT cells could provide better treatments in the future, contributing significantly to the realization of off-the-shelf immune cell therapy. Direct comparisons among the non-armed iγδT cells presented in this study, CAR T cell approaches, and iγδT cells incorporating other armoring strategies will be important subjects for future investigations.

RESOURCE AVAILABILITY

Lead contact

Further information and requests for resources and reagents should be directed to and will be fulfilled by the corresponding author, Takashi Aoi (E-mail: takaaoi@med.kobe-u.ac.jp).

Materials availability

This study did not generate any new unique reagents.

Data and code availability

This study did not generate or analyze any datasets or code.

ACKNOWLEDGMENTS

We thank all members of the laboratory for their scientific discussions and Yoko Matsuoka for administrative support. We are grateful to Dr. Ryoji Yao (Cancer Institute, Japanese Foundation for Cancer Research) for kindly sharing the protocol and offering technical advice regarding the generation of patient-derived colorectal cancer organoids. This work was supported by grants from the Research Center Network for Realization of Regenerative Medicine (JP18bm0704005h0003, JP21bm0404051h0003 and JP256f0137011) (to T.A. and M.K.-A.) from the Japan Agency for Medical Research and Development (AMED), the Akira Sakagami Fund for Research and Education, Kobe University Graduate School of Medicine (to T.A. and M.K.-A.) and JSPS KAKENHI Grant (20KK0202) (to T.A.) and JSPS KAKENHI Grant (22H04973) (to T.A.) and JSPS KAKENHI Grant (23K24320) (to T.A.) and JSPS KAKENHI Grant (24K11929) (to T.A.) and JSPS KAKENHI Grant (25K02728) (to T.A.), and the Hyogo Prefecture Health Foundation “Cancer Research Encouragement Award” (to R.F.), and JSPS KAKENHI Grant (24K23383) (to R.F.). The work was supported by the Program for Forming Japan’s Peak Research Universities (J-PEAKS) from the Japan Society for the Promotion of Science (JSPS). This research was supported by JST Program for co-creating startup ecosystem, Grant no. JPMJSF2315, Japan.

AUTHOR CONTRIBUTIONS

R.F. and T.A. conceptualized the study. R.F. wrote the manuscript with assistance from M.K-A., T. A., and T. A. designed the experiments. R.F. conducted the experiments and analyzed the data. R. F., M.K-A., Y.K., and T.A. interpreted data. A.N. contributed to the generation of CAR-expressing iPSCs. D.K. contributed to the differentiation of iγδT cells from iPSCs. M.H., K.H., and Y.K. contributed to the establishment of the patient-derived colorectal cancer organoids. T.A. provided financial support and took responsibility for this study. All the authors have read and approved the final manuscript. Correspondence and requests for materials should be addressed to T.A.

DECLARATION OF INTERESTS

The authors declare no competing interests.

DECLARATION OF GENERATIVE AI AND AI-ASSISTED TECHNOLOGIES IN THE WRITING PROCESS

During the preparation of this work, the author used DeepL (https://www.deepl.com/translator) and ChatGPT-5 (https://chat.openai.com/) in order to translate specific sentences from Japanese to English. After using this tool/service, the author(s) reviewed and edited the content as needed and take(s) full responsibility for the content of the publication.

STAR★METHODS

Detailed methods are provided in the online version of this paper and include the following:

- KEY RESOURCES TABLE
- EXPERIMENTAL MODEL AND STUDY PARTICIPANT DETAILS
  - Human iPSC lines
  - Human cancer cell lines
  - Patient-derived colorectal cancer organoids
  - Mice
- METHOD DETAILS
  - iPSC culture and differentiation of iPS cells into γδT cells
  - Culture of cancer cells
  - Generation of cancer cell lines overexpressing Venus-Akaluc
  - Introduction into a colorectal cancer cell line
  - Introduction into the jurkat leukemia cell Line
  - Generation of patient-derived colorectal cancer organoids overexpressing Venus-Akaluc
  - Flow cytometry
  - Repertoire analysis
  - Cytotoxicity assay
  - Mixed lymphocyte reaction (MLR)
  - ATP assay and bioluminescence intensity assay
  - Subcutaneous transplantation of cancer cell lines and organoids in mice
  - *In vivo* treatment
  - AkaLumine-AkaLuc bioluminescence imaging system (Aka-BLI)
- QUANTIFICATION AND STATISTICAL ANALYSIS
- ADDITIONAL RESOURCES

SUPPLEMENTAL INFORMATION

Supplemental information can be found online at https://doi.org/10.1016/j.stemcr.2026.103018.

Received: October 20, 2025
Revised: June 20, 2026
Accepted: June 22, 2026
Published: July 23, 2026

REFERENCES

Adachi, K., Kano, Y., Nagai, T., Okuyama, N., Sakoda, Y., and Tamada, K. (2018). IL-7 and CCL19 expression in CAR-T cells improves immune cell infiltration and CAR-T cell survival in the tumor. Nat. Biotechnol. *36*, 346–351. https://doi.org/10.1038/nbt.4086.

Alizadeh, D., Wong, R.A., Yang, X., Wang, D., Pecoraro, J.R., Kuo, C.F., Aguilar, B., Qi, Y., Ann, D.K., Starr, R., et al. (2019). IL15 Enhances CAR-T Cell Antitumor Activity by Reducing mTORC1 Activity and Preserving Their Stem Cell Memory Phenotype. Cancer Immunol. Res. *7*, 759–772. https://doi.org/10.1158/2326-6066.CIR-18-0466.

Blank, C.U., Haining, W.N., Held, W., Hogan, P.G., Kallies, A., Lugli, E., Lynn, R.C., Philip, M., Rao, A., Restifo, N.P., et al. (2019). Defining ’T cell exhaustion’. Nat. Rev. Immunol. *19*, 665–674. https://doi.org/10.1038/s41577-019-0221-9.

Böhme, J., and Pilström, B. (1991). Transgenic mice with ectopic expression of alloantigenic MHC molecules–why are they so

different and of how much help are they? Immunol. Rev. *122*, 21–32. https://doi.org/10.1111/j.1600-065x.1991.tb00594.x.

Davey, M.S., Willcox, C.R., Joyce, S.P., Ladell, K., Kasatskaya, S.A., McLaren, J.E., Hunter, S., Salim, M., Mohammed, F., Price, D.A., et al. (2017). Clonal selection in the human Vdelta1 T cell repertoire indicates gammadelta TCR-dependent adaptive immune surveillance. Nat. Commun. *8*, 14760. https://doi.org/10.1038/ncomms14760.

Fujii, M., Matano, M., Toshimitsu, K., Takano, A., Mikami, Y., Nishikori, S., Sugimoto, S., and Sato, T. (2018). Human Intestinal Organoids Maintain Self-Renewal Capacity and Cellular Diversity in Niche-Inspired Culture Condition. Cell Stem Cell *23*, 787–793.e786. https://doi.org/10.1016/j.stem.2018.11.016.

Fujii, M., Shimokawa, M., Date, S., Takano, A., Matano, M., Nanki, K., Ohta, Y., Toshimitsu, K., Nakazato, Y., Kawasaki, K., et al. (2016). A Colorectal Tumor Organoid Library Demonstrates Progressive Loss of Niche Factor Requirements during Tumorigenesis. Cell Stem Cell *18*, 827–838. https://doi.org/10.1016/j.stem.2016.04.003.

Holtmeier, W., and Kabelitz, D. (2005). gammadelta T cells link innate and adaptive immune responses. Chem. Immunol. Allergy *86*, 151–183. https://doi.org/10.1159/000086659.

Hurton, L.V., Singh, H., Najjar, A.M., Switzer, K.C., Mi, T., Maiti, S., Olivares, S., Rabinovich, B., Huls, H., Forget, M.A., et al. (2016). Tethered IL-15 augments antitumor activity and promotes a stem-cell memory subset in tumor-specific T cells. Proc. Natl. Acad. Sci. USA *113*, E7788–E7797. https://doi.org/10.1073/pnas.1610544113.

Iriguchi, S., Yasui, Y., Kawai, Y., Arima, S., Kunitomo, M., Sato, T., Ueda, T., Minagawa, A., Mishima, Y., Yanagawa, N., et al. (2021). A clinically applicable and scalable method to regenerate T-cells from iPSCs for off-the-shelf T-cell immunotherapy. Nat. Commun. *12*, 430. https://doi.org/10.1038/s41467-020-20658-3.

Izumi, T., Kondo, M., Takahashi, T., Fujieda, N., Kondo, A., Tamura, N., Murakawa, T., Nakajima, J., Matsushita, H., and Kakimi, K. (2013). Ex vivo characterization of gammadelta T-cell repertoire in patients after adoptive transfer of Vgamma9Vdelta2 T cells expressing the interleukin-2 receptor beta-chain and the common gamma-chain. Cytotherapy *15*, 481–491. https://doi.org/10.1016/j.jcyt.2012.12.004.

Kennedy, M., Awong, G., Sturgeon, C.M., Ditadi, A., LaMotte-Mohs, R., Zúñiga-Pflücker, J.C., and Keller, G. (2012). T lymphocyte potential marks the emergence of definitive hematopoietic progenitors in human pluripotent stem cell differentiation cultures. Cell Rep. *2*, 1722–1735. https://doi.org/10.1016/j.celrep.2012.11.003.

Maeda, T., Nagano, S., Ichise, H., Kataoka, K., Yamada, D., Ogawa, S., Koseki, H., Kitawaki, T., Kadowaki, N., Takaori-Kondo, A., et al. (2016). Regeneration of CD8alphabeta T Cells from T-cell-Derived iPSC Imparts Potent Tumor Antigen-Specific Cytotoxicity. Cancer Res. *76*, 6839–6850. https://doi.org/10.1158/0008-5472.CAN-16-1149.

Martinez, M., and Moon, E.K. (2019). CAR T Cells for Solid Tumors: New Strategies for Finding, Infiltrating, and Surviving in the Tumor Microenvironment. Front. Immunol. *10*, 128. https://doi.org/10.3389/fimmu.2019.00128.

Minagawa, A., Yoshikawa, T., Yasukawa, M., Hotta, A., Kunitomo, M., Iriguchi, S., Takiguchi, M., Kassai, Y., Imai, E., Yasui, Y., et al. (2018). Enhancing T Cell Receptor Stability in Rejuvenated iPSC-Derived T Cells Improves Their Use in Cancer Immunotherapy. Cell Stem Cell *23*, 850–858.e4. https://doi.org/10.1016/j.stem.2018.10.005.

Michaels, Y.S., Edgar, J.M., Major, M.C., Castle, E.L., Zimmerman, C., Yin, T., Hagner, A., Lau, C., Hsu, H.H., Ibañez-Rios, M.I., et al. (2022). DLL4 and VCAM1 enhance the emergence of T cell–competent hematopoietic progenitors from human pluripotent stem cells. Sci. Adv. *8*, eabn5522.

Murai, N., Koyanagi-Aoi, M., Terashi, H., and Aoi, T. (2023). Regeneration of cytotoxic gammadeltaT cells with distinctive signatures from human gammadeltaT-derived iPSCs. Stem Cell Rep. *18*, 853–868. https://doi.org/10.1016/j.stemcr.2023.02.010.

Nicol, A.J., Tokuyama, H., Mattarollo, S.R., Hagi, T., Suzuki, K., Yokokawa, K., and Nieda, M. (2011). Clinical evaluation of autologous gamma delta T cell-based immunotherapy for metastatic solid tumours. Br. J. Cancer *105*, 778–786. https://doi.org/10.1038/bjc.2011.293.

Nishimura, T., Kaneko, S., Kawana-Tachikawa, A., Tajima, Y., Goto, H., Zhu, D., Nakayama-Hosoya, K., Iriguchi, S., Uemura, Y., Shimizu, T., et al. (2013). Generation of rejuvenated antigen-specific T cells by reprogramming to pluripotency and redifferentiation. Cell Stem Cell *12*, 114–126. https://doi.org/10.1016/j.stem.2012.11.002.

Odaira, K., Kimura, S.N., Fujieda, N., Kobayashi, Y., Kambara, K., Takahashi, T., Izumi, T., Matsushita, H., and Kakimi, K. (2016). CD27(-)CD45(+) gammadelta T cells can be divided into two populations, CD27(-)CD45(int) and CD27(-)CD45(hi) with little proliferation potential. Biochem. Biophys. Res. Commun. *478*, 1298–1303. https://doi.org/10.1016/j.bbrc.2016.08.115.

Organization, W.H. (2024). https://www.who.int/news-room/fact-sheets/detail/colorectal-cancer.

Pauken, K.E., Sammons, M.A., Odorizzi, P.M., Manne, S., Godec, J., Khan, O., Drake, A.M., Chen, Z., Sen, D.R., Kurachi, M., et al. (2016). Epigenetic stability of exhausted T cells limits durability of reinvigoration by PD-1 blockade. Science *354*, 1160–1165.

Papadopoulou, M., Sanchez Sanchez, G., and Vermijlen, D. (2020). Innate and adaptive gammadelta T cells: How, when, and why. Immunol. Rev. *298*, 99–116. https://doi.org/10.1111/imr.12926.

Park, S.H., Lei, L., D'Souza, D., Zipkin, R., DiMartini, E.T., Atzampou, M., Lallow, E.O., Shan, J.W., Zahn, J.D., Shreiber, D.I., et al. (2023). Efficient electrospray deposition of surfaces smaller than the spray plume. Nat. Commun. *14*, 4896. https://doi.org/10.1038/s41467-023-40638-7.

Rosenberg, S.A., and Restifo, N.P. (2015). Adoptive cell transfer as personalized immunotherapy for human cancer. Science *348*, 62–68. https://doi.org/10.1126/science.aaa4967.

Saoudi González, N., Salvà, F., Ros, J., Baraibar, I., Rodríguez-Castells, M., García, A., Alcaráz, A., Vega, S., Bueno, S., Tabernero, J., and Elez, E. (2023). Unravelling the Complexity of Colorectal Cancer: Heterogeneity, Clonal Evolution, and Clinical Implications. Cancers (Basel) *15*, 4020. https://doi.org/10.3390/cancers15164020.

Sato, K., Koyanagi-Aoi, M., Uehara, K., Yamashita, Y., Shinohara, M., Lee, S., Reinhardt, A., Woltjen, K., Chiba, K., Miyake, H., et al. (2025). Efficient differentiation of human iPSCs into Leydig-like cells capable of long-term stable secretion of testosterone. Stem Cell Rep. *20*, 102392. https://doi.org/10.1016/j.stemcr.2024.102392.

Sen, D.R., Kaminski, J., Barnitz, R.A., Kurachi, M., Gerdemann, U., Yates, K.B., Tsao, H.W., Godec, J., LaFleur, M.W., Brown, F.D., et al. (2016). The epigenetic landscape of T cell exhaustion. Science *354*, 1165–1169. https://doi.org/10.1126/science.aae0491.

Silva-Santos, B., Serre, K., and Norell, H. (2015). gammadelta T cells in cancer. Nat. Rev. Immunol. *15*, 683–691. https://doi.org/10.1038/nri3904.

Stary, V., Pandey, R.V., List, J., Kleissl, L., Deckert, F., Kabiljo, J., Laengle, J., Gerakopoulos, V., Oehler, R., Watzke, L., et al. (2024). Dysfunctional tumor-infiltrating Vdelta1 + T lymphocytes in microsatellite-stable colorectal cancer. Nat. Commun. *15*, 6949. https://doi.org/10.1038/s41467-024-51025-1.

Themeli, M., Kloss, C.C., Ciriello, G., Fedorov, V.D., Perna, F., Gonen, M., and Sadelain, M. (2013). Generation of tumor-targeted human T lymphocytes from induced pluripotent stem cells for cancer therapy. Nat. Biotechnol. *31*, 928–933. https://doi.org/10.1038/nbt.2678.

Timmermans, F., Velghe, I., Vanwalleghem, L., De Smedt, M., Van Coppernolle, S., Taghon, T., Moore, H.D., Leclercq, G., Langerak, A.W., Kerre, T., et al. (2009). Generation of T cells from human embryonic stem cell-derived hematopoietic zones. J. Immunol. *182*, 6879–6888. https://doi.org/10.4049/jimmunol.0803670.

Toshimitsu, K., Takano, A., Fujii, M., Togasaki, K., Matano, M., Takahashi, S., Kanai, T., and Sato, T. (2022). Organoid screening reveals epigenetic vulnerabilities in human colorectal cancer. Nat. Chem. Biol. *18*, 605–614. https://doi.org/10.1038/s41589-022-00984-x.

Trotman-Grant, A.C., Mohtashami, M., De Sousa Casal, J., Martinez, E.C., Lee, D., Teichman, S., Brauer, P.M., Han, J., Anderson, M.K., and Zúñiga-Pflücker, J.C. (2021). DL4-mubeads induce T cell lineage differentiation from stem cells in a stromal cell-free system. Nat. Commun. *12*, 5023. https://doi.org/10.1038/s41467-021-25245-8.

Tsiverioti, C.A., Gottschlich, A., Trefny, M., Theurich, S., Anders, H.J., Kroiss, M., and Kobold, S. (2024). Beyond CAR T cells: exploring alternative cell sources for CAR-like cellular therapies. Biol. Chem. *405*, 485–515. https://doi.org/10.1515/hsz-2023-0317.

Ueda, T., Shiina, S., Iriguchi, S., Terakura, S., Kawai, Y., Kabai, R., Sakamoto, S., Watanabe, A., Ohara, K., Wang, B., et al. (2023). Optimization of the proliferation and persistency of CAR T cells derived from human induced pluripotent stem cells. Nat. Biomed. Eng. *7*, 24–37. https://doi.org/10.1038/s41551-022-00969-0.

Vantourout, P., and Hayday, A. (2013). Six-of-the-best: unique contributions of gammadelta T cells to immunology. Nat. Rev. Immunol. *13*, 88–100. https://doi.org/10.1038/nri3384.

Vizcardo, R., Masuda, K., Yamada, D., Ikawa, T., Shimizu, K., Fujii, S.I., Koseki, H., and Kawamoto, H. (2013). Regeneration of human tumor antigen-specific T cells from iPSCs derived from mature CD8(+) T cells. Cell Stem Cell *12*, 31–36. https://doi.org/10.1016/j.stem.2012.12.006.

Wakao, H., Yoshikiyo, K., Koshimizu, U., Furukawa, T., Enomoto, K., Matsunaga, T., Tanaka, T., Yasutomi, Y., Yamada, T., Minakami, H., et al. (2013). Expansion of functional human mucosal-associated invariant T cells via reprogramming to pluripotency and redifferentiation. Cell Stem Cell *12*, 546–558. https://doi.org/10.1016/j.stem.2013.03.001.

Watanabe, D., Koyanagi-Aoi, M., Taniguchi-Ikeda, M., Yoshida, Y., Azuma, T., and Aoi, T. (2018). The Generation of Human gammadeltaT Cell-Derived Induced Pluripotent Stem Cells from Whole Peripheral Blood Mononuclear Cell Culture. Stem Cells Transl. Med. *7*, 34–44. https://doi.org/10.1002/sctm.17-0021.

Wei, J., Long, L., Zheng, W., Dhungana, Y., Lim, S.A., Guy, C., Wang, Y., Wang, Y.D., Qian, C., Xu, B., et al. (2019). Targeting REGNASE-1 programs long-lived effector T cells for cancer therapy. Nature *576*, 471–476. https://doi.org/10.1038/s41586-019-1821-z.

Wherry, E.J., Ha, S.J., Kaech, S.M., Haining, W.N., Sarkar, S., Kalia, V., Subramaniam, S., Blattman, J.N., Barber, D.L., and Ahmed, R. (2007). Molecular signature of CD8+ T cell exhaustion during chronic viral infection. Immunity *27*, 670–684. https://doi.org/10.1016/j.immuni.2007.09.006.

Wherry, E.J., and Kurachi, M. (2015). Molecular and cellular insights into T cell exhaustion. Nat. Rev. Immunol. *15*, 486–499. https://doi.org/10.1038/nri3862.

Yu, L., Wang, Z., Hu, Y., Wang, Y., Lu, N., and Zhang, C. (2023). Tumor-infiltrating gamma delta T-cells reveal exhausted subsets with remarkable heterogeneity in colorectal cancer. Int. J. Cancer *153*, 1684–1697. https://doi.org/10.1002/ijc.34669.

Yuan, L., Ma, X., Yang, Y., Qu, Y., Li, X., Zhu, X., Ma, W., Duan, J., Xue, J., Yang, H., et al. (2023). Phosphoantigens glue butyrophilin 3A1 and 2A1 to activate Vgamma9Vdelta2 T cells. Nature *621*, 840–848. https://doi.org/10.1038/s41586-023-06525-3.

Zhang, P., Zhang, G., and Wan, X. (2023). Challenges and new technologies in adoptive cell therapy. J. Hematol. Oncol. *16*, 97. https://doi.org/10.1186/s13045-023-01492-8.

Zhao, Y., Chen, J., Andreatta, M., Feng, B., Xie, Y.Q., Wenes, M., Wang, Y., Gao, M., Hu, X., Romero, P., et al. (2024). IL-10-expressing CAR T cells resist dysfunction and mediate durable clearance of solid tumors and metastases. Nat. Biotechnol. *42*, 1693–1704. https://doi.org/10.1038/s41587-023-02060-8.

STAR★METHODS

KEY RESOURCES TABLE

REAGENT or RESOURCE	SOURCE	IDENTIFIER
**Antibodies**		
anti-human CD3-PC7	eBiosciences	Clone: UCHT1, Cat: 25-0038
anti-human CD7-FITC	eBiosciences	Clone: 4H9, Cat: 17-0078
anti-human CD5-PE	eBiosciences	Clone: VCHT2, Cat: 12-0059
anti-human CD335-PE	BioLegend	Clone:9E2, Cat:331907
anti-human γδTCR-PE	eBiosciences	Clone: B11, Cat: 12-9959
anti-human CD3-eFluorTM450	eBiosciences	Clone: OKT3, Cat: 48-0037-42
anti-human CD4-FITC	eBiosciences	Clone: OKT4, Cat: 11-0048-42
anti-human CD8α-PE	eBiosciences	Clone: OKT8, Cat: 12-0086-42
anti-human CD8β-PC7	eBiosciences	Clone: SIDI8BEE, Cat: 25-5273-41
anti-human CD314-FITC	BioLegend	Clone: P30-15, Cat: 325213
anti-human CD226-PE	BioLegend	Clone: TX25, Cat: 338305
anti-human CD45RA-FITC	eBiosciences	Clone: HI100, Cat: 11-0458-41
anti-human CD27-PE	BioLegend	Clone: O323, Cat: 302807
anti-human Perforin-FITC	BioLegend	Clone: dG9, Cat: 308103
anti-human Granzyme B-PE	eBiosciences	Clone: GB11, Cat: 12-8899-41
anti-human IFN-γ-PC7	BioLegend	Clone: 4 S. B3, Cat: 502527
pan-cytokeratin	Abcam	Clone: AE1/AE3, Cat: ab27988;
CD3	Abcam	Clone: F7.2.38, Cat: ab17143
γδTCR	Thermo Fisher Scientific	Clone: 5A6. E9, Cat: TCR1061
**Chemicals, peptides, and recombinant proteins**		
StemFit AK02N	Ajinomoto	Cat# AJ100
1*X* TrypLE Select	Thermo Fisher Scientific	Cat# A12859-01
iMatrix-511 silk	Nippi	Cat# 892021
CultureSure Y-27632	FUJIFILM Wako	Cat# 034-24023
CHIR99021	TOCRIS	Cat# 4423
BMP4	R&D Systems	Cat# 314-BP-050
VEGF	R&D Systems	Cat# 293-VE-050
Advanced DMEM/F12	Thermo Fisher Scientific	Cat# 12634010
SB431542	WAKO	Cat# 033-24631
bFGF	WAKO	Cat# 060-04543
IL-3	Peprotech	Cat# AF-200-03

(*Continued on next page*)

Continued

REAGENT or RESOURCE	SOURCE	IDENTIFIER
IL-6	R&D	Cat# 206-IL
FLT3L	R&D	Cat# 308-FK
SCF	R&D	Cat# 255-SC
ESPO	Kyowa Kirin	N/A
StemFit For Differentiation	AJINOMOTO	N/A
VCAM1	R&D Systems	Cat# ADP5
DLL4 protein	Sino Biological Inc.	Cat# 862-VC
StemSpan™ SFEM II	STEMCELL	Cat# ST-09605
10*X* StemSpan™ Lymphoid Progenitor Expansion Supplement	STEMCELL	Cat# 09915
DKK-1	R&D	Cat# 5439-DK
azelaic acid	FUJIFILM Wako	Cat# 123-99-9
RPMI1640	Nacalai Tesque	Cat# 30264
IL-15	WAKO	Cat# 095-07031
2DG	FUJIFILM Wako	Cat# 154-17-6
HMBPP	Cayman Chemical Company	Cat# 13580
HEPES	Thermo Fisher Scientific	Cat# 15630080
sodium pyruvate	Thermo Fisher Scientific	Cat# 11360070
100*X* GlutaMax	Thermo Fisher Scientific	Cat# 35050061
Tks Gflex™ DNA Polymerase	TaKaRa	Cat# R060A
LR Clonase II	Invitrogen	Cat# 11791020
Lipofectamine™ 3000	Invitrogen	Cat# L3000015
Opti-MEM® I Reduced Serum Medium	Invitrogen	Cat# 31985-062
P3000 Reagent	Invitrogen	Cat# L3000015
Liberase TH Research Grade	Roche	Cat# 5401135001
Growth Factor Reduced Matrigel	Merck	Cat# CLS354230
B-27 Supplement	Thermo Fisher Scientific	Cat# 12587001
Primocin	InvivoGen	Cat# ant-pm-05
gastrin I	Sigma-Aldrich	Cat# SCP0152
recombinant human EGF	R&D	Cat# 236-EG
recombinant human Noggin	(R&D	Cat# 6057-NG
N-acetylcysteine	WAKO	Cat# 616-91-1
recombinant human IGF-1	BioLegend	Cat# 588604
recombinant human FGF-basic	WAKO	Cat# 587-97873
FuGENE HD	Roche	Cat# E2311

(*Continued on next page*)

Continued

REAGENT or RESOURCE	SOURCE	IDENTIFIER
Fetal Bovine Serum (FBS)	Biowest	Cat# S1650
carboxyfluorescein diacetate succinimidyl ester (CFSE)	Thermo Fisher Scientific	Cat# 65-0850-84
7AAD	ISP	Cat# 7AAD
AkaLumine-HCl	FUJIFILM Wako Pure Chemical Corporation	Cat# 018-26703
A83-01	Sigma-Aldrich	Cat# SML0788
ezDNase	Thermo Fisher Scientific	Cat# 11766051
**Experimental models: Cell lines**		
Human γδT-iPSC clone 62B3	Watanabe et al., 2018	N/A
Human γδT-iPSC clone 121-3	Murai et al., 2023	N/A
CAR-expressing γδT-iPSC line	This study	N/A
Jurkat cells	Laboratory stock	RRID:CVCL_0367
JurkatA cells	This study	Venus-Akaluc-expressing Jurkat cells
SW480 cells	Laboratory stock	RRID:CVCL_0546
SW480A cells	This study	SW480 cells expressing Venus-Akaluc
Rm8A colorectal cancer organoids	This study |	N/A
Rm19 A colorectal cancer organoids	This study	N/A
**Biological samples**		
Human PBMCs from healthy donors	Kobe University	N/A
Patient-derived rectal tumor tissue for Rm8 organoids	Kobe University Hospital	N/A
Patient-derived rectal tumor tissue for Rm19 organoids	Kobe University Hospital	N/A
**Critical commercial assays**		
ezDNase	Thermo Fisher Scientific	Cat# 11766051
CellTiter-Glo®	Promega	Cat# G7570
**Experimental models: Organisms/strains**		
NSG mice (NOD.Cg-Prkdcscid Il2rgtm1Wjl/SzJ)	The Jackson Laboratory Japan	RRID:IMSR_JAX:005557
**Recombinant DNA**		
pHULK-PB-CAR-CD19-BBζ	This study	N/A
pHULK-PB-Venus-Akaluc	This study	N/A
pENTR™/D-TOPO™	Thermo Fisher Scientific	Cat# K240020SP
pcDNA3 Venus-Akaluc	RIKEN	Cat# RDB15781
pMXs-Venus-Akaluc	Sato et al.	N/A
pSLCAR-CD19-BBζ	Addgene	Cat#135992
**Other**		
BD FACSMelody™ Cell Sorter	BD Biosciences	N/A
NEPA21 Type II	NEPAGENE	N/A

(*Continued on next page*)

Continued		
REAGENT or RESOURCE	SOURCE	IDENTIFIER
RF-500	Sysmex	N/A
CytoFLEX	BECKMAN COULTER	N/A
GloMax® Discover/Explorer System	Promega	N/A
xCELLigence Real-time Cell Analyzer (RTCA) SP system	ACEA BioSciences	Cat# 00380601030
IVIS Lumina LT imaging system	PerkinElmer	N/A

EXPERIMENTAL MODEL AND STUDY PARTICIPANT DETAILS

Human iPSC lines

Peripheral blood Vγ9Vδ2 T cell-derived hiPSC clones 62B3 and 121–3 were used in this study. These hiPSC lines were established from peripheral blood mononuclear cells obtained from healthy volunteers after informed consent, as previously described. The use of human samples was approved by the Institutional Review Board of Kobe University Graduate School of Medicine. The sex of the donor cells was not used as an experimental variable in this study.

Human cancer cell lines

Jurkat and SW480 cells were used as target cancer cell lines. Venus-Akaluc-expressing JurkatA and SW480A cells were generated for fluorescence and bioluminescence-based assays. Cells were maintained under standard mammalian cell culture conditions at 37°C in a humidified incubator with 5% CO_2. Available authentication information and RRIDs are provided in the Key Resources Table.

Patient-derived colorectal cancer organoids

Patient-derived colorectal cancer organoids Rm8 and Rm19 were established from surgically resected rectal tumor tissues obtained at Kobe University Hospital. All experiments using human tumor tissues were approved by the Ethics Committee of Kobe University, and informed consent was obtained from the patients. Organoids were maintained in Matrigel under colorectal cancer organoid culture conditions as described in the Method Details section.

Mice

Immunodeficient NSG mice were used for xenograft experiments. Female mice aged 4–8 weeks were used in all the experiments. JurkatA cells, SW480A cells, or patient-derived colorectal cancer organoids were transplanted subcutaneously, and iγδT cells were administered either locally or intravenously depending on the experimental design. Mice were maintained under specific pathogen-free conditions in accordance with institutional guidelines. All animal experiments were approved by the relevant animal experiment committee of Kobe University.

METHOD DETAILS

iPSC culture and differentiation of iPS cells into γδT cells

We cultured peripheral blood Vγ9Vδ2 T cell -derived hiPSC clones 62B3 and 121–3 as previously described(Murai et al., 2023; Watanabe et al., 2018). A CAR-expressing γδT-iPS cell line (Figure S1B) was established by transfecting the pHULK-PB-CAR-CD19-BBζ into 121–3. The pHULK-PB-CAR-CD19-BBζ plasmid was generated using the Gateway cloning system with the plasmid pSLCAR-CD19-BBζ (Addgene #135992) as the template. Details of the pHULK-PB vector are provided in the “Generation of cancer cell lines overexpressing Venus-Akaluc” section described later in discussion. Differentiation of these iPSCs into γδT cells was performed as follows.

In a previous report(Murai et al., 2023), human γδT-iPSCs were seeded at a density of 2.0×10^3 cells per well in a 6-well plate, seven days before induction. In this study, we used cells seeded at a density of 1.0×10^3 cells per well in a 6-well plate.

Day 0: The medium was completely replaced with StemFit AK02N (AJINOMOTO, Tokyo, Japan), supplemented with 4 μM CHIR99021 (Tocris, 4423), 80 ng/mL BMP4 (R&D, 314-BP), and 80 ng/mL vascular endothelial growth factor (VEGF) (R&D, 293-VE).

Day 2: The medium was replaced with Advanced DMEM/F12 (Thermo Fisher Scientific, 12634010) supplemented with 2 mM L-glutamine, 2 μM SB431542 (WAKO, 033–24631), 50 ng/mL bFGF (WAKO, 060–04543), 50 ng/mL SCF (R&D, 255-SC), 80 ng/mL VEGF, and 20% StemFit For Differentiation (AJINOMOTO, Tokyo, Japan).Day 4: The medium was replaced with Advanced DMEM/F12 supplemented with 2 mM L-glutamine, 50 ng/mL IL-3 (Peprotech, AF-200-03), 50 ng/mL IL-6 (R&D, 206-IL), 50 ng/mL FLT3L (R&D, 308-FK), 50 ng/mL SCF, 20 ng/mL VEGF, 10 IU/mL EPO (ESPO, Kyowa Kirin), and 20% StemFit for Differentiation.

On days 6 and 8, the medium was replaced with Advanced DMEM/F12 supplemented with 2 mM L-glutamine, 50 ng/mL IL-6, 50 ng/mL SCF, 10 IU/mL EPO, and 20% StemFit for Differentiation.

Day 10: The adherent cells were detached by pipetting and incubated at 37°C for 20 min. The supernatant was filtered through a 100 μm cell strainer, followed by a 30 μm cell strainer. After centrifugation at 1,200 rpm for 5 min, cells were seeded into 48-well plates at 1.2×10^4 cells per well. Plates were pre-coated with a carbonate-bicarbonate buffer containing 5 μg/mL VCAM1 (R&D Systems, ADP5) and 10 μg/mL recombinant human DLL4 protein (Sino Biological Inc., 862-VC) 24 h before cell seeding. The medium used was StemSpan SFEM II (STEMCELL, ST-09605) supplemented with 10% 10*X* StemSpan Lymphoid Progenitor Expansion Supplement (STEMCELL, ST-09915), 30 ng/mL DKK-1 (R&D, 5439-DK), and 5 mM azelaic acid (FUJIFILM Wako, 123-99-9). Half of the medium was replaced twice a week until the next step.

Day 24: Half of the medium was replaced with RPMI1640 (Nacalai Tesque, 30264) supplemented with 100 ng/mL IL-15 (FUJIFILM Wako, 095–07031), 1 mM 2DG (FUJIFILM Wako, 154-17-6), 1 nM HMBPP (Cayman Chemical Company, 13580), 10 mM HEPES (Thermo Fisher Scientific, 15630080), 1 mM sodium pyruvate (Thermo Fisher Scientific, 11360070), 1% 100*X* GlutaMax (Thermo Fisher Scientific, 35050061), and 20% StemFit for Differentiation. Half-medium changes were continued twice per week thereafter. Cells were used for experiments after one week or more of stimulation.

Culture of cancer cells

The Jurkat cancer cell lines were obtained from Riken Bio Resource Center (Tsukuba, Ibaraki, Japan). The SW480 cancer cell line was obtained from American Type Culture Collection ([ATCC] Manassas, VA). Jurkat cells (Riken BRC, RCB0806) were cultured in RPMI 1640 containing 10% FBS and were passaged every 3 days. The SW480 (ATCC, CCL-228) cell lines were cultured in DMEM (Nacalai tesque, 08458–45) containing 10% FBS. They were passaged every 3–5 days.

Generation of cancer cell lines overexpressing Venus-Akaluc

Jurkat and SW480 cancer cell lines were cultured as previously described(Murai et al., 2023). Venus-Akaluc-overexpressing cells were generated by stable transfection with pHULK-PB-Venus-Akaluc vector. The plasmid pHULK-PB-Venus-Akaluc was constructed as follows:

Venus-Akaluc cDNA was amplified from the pcDNA3 Venus-Akaluc vector using Tks Gflex DNA Polymerase (TaKaRa, R060A) and cloned into pENTR/D-TOPO (Thermo Fisher Scientific K240020SP) to create pENTR-Venus-Akaluc. The pcDNA3 Venus-Akaluc plasmid was provided by RIKEN BRC through the National BioResource Project (NBRP) of MEXT (Cat. RDB15781). The primers used for PCR amplification were as follows: FW, 5′-CACCA TGGTG AGCAA GGG-3′ and RV, 5′-TTACA CGGCG ATCTT GC-3′. The PB-GFP plasmid (pHULK PiggyBac Mammalian Expression Vector-CometGFP, PJ509-02) was purchased from DNA2.0. The gateway (gw) cassette was cloned into the BsaI sites of the PB-GFP plasmid (GFP was replaced with a gw cassette). The product pHULK-PB-gw was used for gateway cloning. The Venus-Akaluc cDNA fragment was mobilized from the pENTR-Venus-Akaluc clone into pHULK-PB-gw using LR Clonase II (Invitrogen, 11791020) to produce pHULK-PB-Venus-Akaluc.

Introduction into a colorectal cancer cell line

Three days prior to transfection, SW480 cells (RRID: CVCL_0546) were seeded in a 6-well plate at a density of 5×10^5 cells/well to reach 70–90% confluency on the day of transfection. Lipofectamine 3000 (Invitrogen, L3000015) was diluted by adding 7.5 μL–125 μL Opti-MEM I Reduced Serum Medium (Invitrogen, #31985–062). Separately, 5 μg of plasmid DNA was mixed with 250 μL Opti-MEM I Reduced Serum Medium and 10 μL P3000 Reagent (Invitrogen, L3000015). Diluted Lipofectamine 3000 was added to the plasmid mixture in 125 μL increments. The resulting mixture was added at 250 μL per well for transfection.

To enrich the cell population with high Venus expression, sorting was performed using a BD FACSMelody Cell Sorter (BD Biosciences).

Introduction into the jurkat leukemia cell Line

Three days prior to transfection, Jurkat cells were seeded in a 6 cm dish at a density of 1×10^6 cells. On the day of transfection, the cells were collected along with the culture medium, centrifuged at $160 \times g$ for 5 min, and the supernatant was removed. The cell pellet was suspended in 100 μL Opti-MEM I Reduced Serum Medium containing 5 μg plasmid DNA. Electroporation was performed at 125 V using NEPA21 Type II (NEPAGENE).

From Day 2, drug selection was initiated with 2 μg/mL puromycin, resulting in a Venus expression rate exceeding 95%.

Generation of patient-derived colorectal cancer organoids overexpressing Venus-Akaluc

Colorectal cancer organoids were derived from patients who had undergone surgical resection of rectal tumors at Kobe University Hospital. All experiments were conducted with the approval of the Ethics Committee of Kobe University. The culture process was slightly modified from methods described in the literature(Toshimitsu et al., 2022; Fujii et al., 2016; Fujii et al., 2018). Briefly, tumor samples were minced and incubated with 50 μg/mL Liberase TH Research Grade (Roche, 5401135001) and 10 μM Y-27632 (WAKO) at 37°C for 30 min, with pipetting every 10 min during the incubation. The residual tissue fragments were further incubated with TrypLE Select (Thermo Fisher Scientific) at 37°C for 10 min. Isolated tumor cells were suspended in Matrigel (growth factor-reduced, Corning, Merck, CLS354230) and seeded at 50 μL per well in a 24-well plate. After the Matrigel solidified, the following culture media were added: Advanced DMEM/F12 (Thermo Fisher Scientific) supplemented with Primocin (InvivoGen, ant-pm-05), 10 mM HEPES (Thermo Fisher Scientific), 1*X* GlutaMAX (Thermo Fisher Scientific), 1*X* B-27 Supplement (Thermo Fisher Scientific, 12587001), 10 nM gastrin I (Sigma-Aldrich, SCP0152), 1 mM N-acetylcysteine (WAKO, 616-91-1), 50 ng/mL recombinant human EGF (R&D, 236-EG), 100 ng/mL recombinant human Noggin (R&D, 6057-NG), 500 nM A83-01 (Sigma-Aldrich, SML0788), 100 ng/mL recombinant human IGF-1 (BioLegend, 588604), and 50 ng/mL recombinant human FGF-basic (FGF-2) (WAKO, 587–97873). During the first 48 h, 10 μM Y-27632 (WAKO) was added to the culture medium.

Next, we generated colorectal cancer organoids that overexpressed Venus-Akaluc via retroviral transduction. The pMXs-Venus-Akaluc plasmid vector(Sato et al., 2025) was transfected into packaging cells (PLAT-A) using FuGENE HD (Roche) according to the manufacturer's instructions. The medium was replaced the day after transfection, and on the second day, the culture supernatant was collected and filtered through a 0.45 μm filter. Polybrene was added to the filtered supernatant at a final concentration of 8 μg/mL.

The organoids (Rm8 and Rm19) were cultured on collagen-coated plates for two days before infection. Four days after infection, organoids were transferred to Matrigel-coated plates. The expression of green Venus fluorescent protein was observed using a fluorescence microscope two days after retroviral infection. For Rm8 organoids, fluorescent protein-expressing cells were purified using a BD FACSMelody Cell Sorter (BD Biosciences).

Flow cytometry

Flow cytometry (FCM) was performed using the RF-500 (Sysmex) for Figures 1B and 2F, BD FACSMelody Cell Sorter (BD Biosciences) for Figures 1C and S1A, and CytoFLEX (BECKMAN COULTER) for Figures 1B and 1D. Differentiated cells were incubated with diluted antibodies at 4°C in the dark for 20 min. The following antibodies were used: anti-human CD3-PC7 (1:50; eBiosciences Clone: UCHT1, Cat: 25–0038), anti-human CD7-FITC (1:50, eBiosciences Clone: 4H9, Cat: 17–0078), anti-human CD5-PE (1:50, eBiosciences Clone: VCHT2, Cat: 12–0059), anti-human CD335-PE (1:50; BioLegend Clone:9E2, Cat:331907), and anti-human γδTCR-PE (1:50; eBiosciences Clone: B11, Cat: 12–9959), anti-human CD3-eFluorTM450 (1:50, eBiosciences Clone: OKT3, Cat: 48-0037-42), anti-human CD4-FITC (1:50, eBiosciences Clone: OKT4, Cat: 11-0048-42), anti-human CD8α-PE (1:50, eBiosciences Clone: OKT8, Cat: 12-0086-42), anti-human CD8β-PC7 (1:50, eBiosciences Clone: SIDI8BEE, Cat: 25-5273-41), anti-human CD314-FITC (1:50, BioLegend Clone: 1D11, Cat: 320819), anti-human CD337-PC7 (1:50, BioLegend Clone: P30-15, Cat: 325213), anti-human CD226-PE (1:50, BioLegend Clone: TX25, Cat: 338305), anti-human CD45RA-FITC (1:50, eBiosciences Clone: HI100, Cat: 11-0458-41), anti-human CD27-PE (1:50, BioLegend Clone: O323, Cat: 302807), anti-human Perforin-FITC (1:50, BioLegend Clone: dG9, Cat: 308103), anti-human Granzyme B-PE (1:50, eBiosciences Clone: GB11, Cat: 12-8899-41), anti-human IFN-γ-PC7 (1:50, BioLegend Clone: 4 S. B3, Cat: 502527). For cytoplasmic CD3 detection, differentiated cells were washed with PBS(−) and fixed with 4% paraformaldehyde for 10 min. After another wash, the cells were permeabilized and incubated with antibodies diluted in 0.3% Triton X-100 containing 1% human serum for 15 min at room temperature under light shielding. After washing, cells were analyzed using an RF-500 flow cytometer. Anti-human CD3-PC7 (1:50, eBioscience Clone: UCHT1, Cat: 25–0038) was used.

Repertoire analysis

A repertoire analysis was performed using iγδT cells (121-3–derived) on day 34 of differentiation. Total RNA was extracted using TRIzol reagent and treated with ezDNase (Invitrogen, Thermo Fisher Scientific, Waltham, MA, USA, 11766051) to remove genomic DNA contamination. A sequencing analysis was outsourced to Takara Bio Inc. (Shiga, Japan).

Cytotoxicity assay

Jurkat cells (transduced with Venus Akaluc) were seeded at 5×10^3 cells per well in a 96-well plate. On the same day (day 0), iγδT cells were added at E:T ratios of 4:1, 2:1, and 1:1. On day 5, 1 μL of 10 mg/mL akalumine was added, and the supernatant was collected. Luminescence was measured using a the GloMax Discover/Explorer System (Promega).

Cytotoxicity against SW480 cells was evaluated using an xCELLigence Real-time Cell Analyzer (RTCA) SP system (ACEA BioSciences, catalog number for complete system: 00380601030). Fifty microliters of medium (10% FBS/DMEM) was added to the wells of an xCELLigence E-Plate (ACEA BioSciences, VIEW 96 PET plate). Before adding 100 μL of SW480A cells (2×10^4 cells/well), the E-Plate was mounted on the xCELLigence system to record the background measurements for each well. After allowing the cells to settle for 30 min at room temperature, the E-Plate was reinstalled in the xCELLigence analyzer and incubated at 37°C with 5% CO_2 for 24 h.

After 24 h, 100 μL of the culture supernatant was removed, and iγδT cells were added at E:T ratios of 2:1 and 1:1, adjusted to a total volume of 100 μL. After allowing the plate to settle at room temperature for 30 min, it was reinstalled in the xCELLigence analyzer and incubated at 37°C with 5% CO_2 for 48 h. Data from time points close to the 48-h mark were used for the analysis.

Mixed lymphocyte reaction (MLR)

The stimulating cells were iγδT cells, and the responding cells were peripheral blood mononuclear cells (PBMCs). PBMCs were pre-labeled with 0.1 μg/mL carboxyfluorescein diacetate succinimidyl ester (CFSE) (65-0850-84, Thermo Fisher Scientific). Mixed cells (stimulating and responding cells in a ratio of 1:1) were cultured for 7 h. After staining dead cells with 7AAD (ISP, 400TEST), the mixed cells were collected and flow cytometry (FCM) was performed. As controls, PBMCs were cultured under the same conditions and co-cultured with Jurkat cells and iγδT cells under the same conditions.

ATP assay and bioluminescence intensity assay

Organoids are dissociated into single cells and were seeded at 1×10^4 cells per well on collagen-coated 48 well plate (Cell Matrix Type 1-A, reconstitution buffer, RPMI concentrated culture medium). On Day 2 after seeding, iγδT cells were added at an effector-to-target (E:T) ratio of 10:1, and co-culture was continued for 5 days.

For the ATP assay, 100 μL of CellTiter-Glo (Promega, G7570) was added to each well and the plate was shaken at room temperature for 30 min to 2 h. Subsequently, 100 μL of the supernatant was collected, and luminescence was measured using the GloMax Discover/Explorer System (Promega).

To measure bioluminescence intensity (BLI), 50 μg/mL Liberase TH Research Grade (Roche) and 1 μL of 10 mg/mL AkaLumine-HCL (FUJIFILM Wako Pure Chemical Corporation, 018–26703) were added to the wells. The plate was shaken at room temperature for 30 min to 2 h. All media in the wells were collected and luminescence was measured using the GloMax Discover/Explorer System (Promega).

Subcutaneous transplantation of cancer cell lines and organoids in mice

NSG mice (NOD.Cg-Prkdcscid Il2rgtm1Wjl/SzJ, RRID:BCBC4611) were purchased from Jackson Laboratories (West Grove, PA, USA). The mice were housed under a 14-h light/dark cycle at a controlled temperature of 24°C with 50% humidity. Animal handling and experimental procedures were approved by the Animal Experiment Committee of Kobe University and conducted in accordance with the relevant Japanese government regulations. The approval number for this study is P220810. Female mice aged 4–8 weeks were used in all the experiments.

Mice were anesthetized with inhaled isoflurane and placed in a prone position. A total of 5×10^5 Venus-Akaluc transduced JurkatA or SW480A cells were mixed with their respective culture media, while 1×10^5 organoid cells (Rm19 A or Rm8A) were mixed with the media and Matrigel to prepare a 200 μL cell suspension. The cell suspension was injected locally into the right dorsal flank of each mouse. The mice were euthanized 4–6 weeks post-transplantation.

In vivo treatment

Local administration. For local administration, iγδT cells were mixed with cancer cell lines or organoids in a tube before subcutaneous injection. The effector-to-target (E:T) ratios were as follows: 2:1 (T = Jurkat), 5:1 (T = SW480), 10:1 (T = Rm19 or Rm8A).

Intravenous administration. For intravenous administration, patient-derived colorectal cancer organoid Rm8A was transplanted subcutaneously, and iγδT cells were administered at an E:T ratio of 10:1 via retroorbital injection at the time of transplantation. Subsequently, the same dose of iγδT cells was administered weekly via retro-orbital injection for four weeks.

For post-tumor engraftment intravenous administration, Rm8A organoids were transplanted subcutaneously and tumor engraftment was confirmed 10 days later using IVIS imaging. iγδT cells, at a dose equivalent to 40 times the number of transplanted organoid cells, were administered via retro-orbital injection weekly for three weeks, and observations were conducted for four weeks.

In one experiment using Jurkat cells (Figures S3A and S3B), three mice were treated with iγδT cells derived from a single differentiation and compared with three control mice. In other *in vivo* experiments, iγδT cells derived from three or more independent differentiations were tested, with $n = 1$ treated mouse and $n = 1$ control mouse assigned to each differentiation experiment. These data were displayed individually and were not subjected to pooled statistical analysis. Therefore, these experiments were used to assess reproducibility across independent differentiations rather than to perform statistical comparisons between treatment groups.

AkaLumine-AkaLuc bioluminescence imaging system (Aka-BLI)

BLI was performed using the IVIS Lumina LT Imaging System (PerkinElmer, Waltham, MA, USA) and an image analysis was conducted using Living Image (ver. 4.3, Revvity). Aka Lumine n-Hydrochloride was administered to the mice via subcutaneous injection at a concentration of 2.5 mg/mL prior to imaging. Measurements were taken 5 min after substrate administration. Image acquisition settings: Exposure time: 10 s, Binning: Medium, 8, XFOV (Field of View): 24 × 24 cm, f/stop value: 1.

Immunohistochemistry. Tissues were fixed in 10% Formalin Neutral Buffer Solution (FUJIFILM) for two days and embedded in paraffin. Paraffin-embedded tissues were sectioned into 5 μm slices. H&E staining was performed according to the standard protocol. For immunostaining, the sections were deparaffinized, rehydrated, and subjected to antigen retrieval. The following primary antibodies were used for immunostaining: pan-cytokeratin (1:100, ab27988; Abcam AE1/AE3, RRID:AB_777047), CD3 (1:100, ab17143, Abcam F7.2.38, RRID:AB_302587), γδTCR (1:100, TCR1061 5A6.E9, RRID:AB_3270111).

QUANTIFICATION AND STATISTICAL ANALYSIS

Statistical analyses were performed using GraphPad Prism 9 (GraphPad Software, San Diego, CA, USA). Details of statistical tests, sample numbers, and definitions of experimental units are provided in the corresponding figure legends.

For *in vitro* experiments, comparisons between groups were performed using unpaired two-tailed Student's t-tests or one-way ANOVA with appropriate multiple-comparison tests as indicated in the figure legends. A p value <0.05 was considered statistically significant.

For *in vivo* experiments evaluating reproducibility across independent differentiations, iγδT cells generated from independent differentiation experiments were assessed individually. In these experiments, each differentiation experiment was treated as an independent experimental unit, and descriptive comparisons are presented without pooled statistical analyses.

ADDITIONAL RESOURCES

This study did not generate a website, online resource, or clinical trial registration relevant to the work described herein.

Stem Cell Reports, Volume *21*

Supplemental Information

Allogeneic iPSC-derived γδT cells demonstrate antitumor efficacy against patient-derived tissues

Ryoko Futai, Michiyo Koyanagi-Aoi, Aito Nakahashi, Daiki Katsura, Manabu Horikawa, Kazumasa Horie, Yoshihiro Kakeji, Yuzo Kodama, and Takashi Aoi

Figure S1

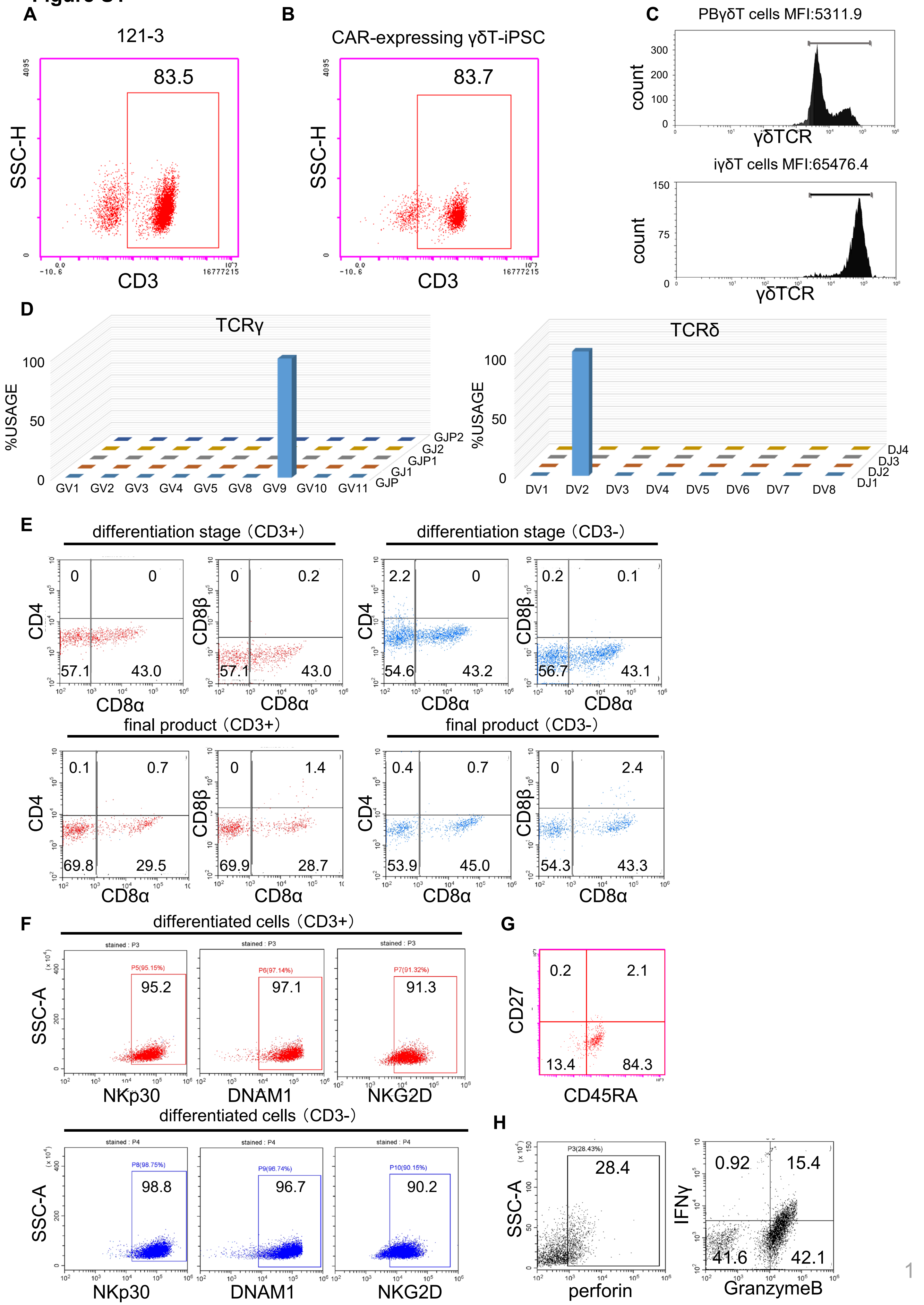

A
121-3
83.5
SSC-H
CD3
B
CAR-expressing γδT-iPSC
83.7
SSC-H
CD3
C
PBγδT cells MFI:5311.9
count
γδTCR
iγδT cells MFI:65476.4
count
γδTCR
D
TCRγ
%USAGE
GV1
GV2
GV3
GV4
GV5
GV8
GV9
GV10
GV11
GJP
GJ1
GJP1
GJ2
GJP2
TCRδ
%USAGE
DV1
DV2
DV3
DV4
DV5
DV6
DV7
DV8
DJ1
DJ2
DJ3
DJ4
E
differentiation stage（CD3+）
differentiation stage（CD3-）
final product（CD3+）
final product（CD3-）
CD4
CD8β
CD8α
F
differentiated cells（CD3+）
differentiated cells（CD3-）
SSC-A
NKp30
DNAM1
NKG2D
95.2
97.1
91.3
98.8
96.7
90.2
G
CD27
CD45RA
0.2
2.1
13.4
84.3
H
SSC-A
perforin
28.4
IFNγ
GranzymeB
0.92
15.4
41.6
42.1

Supplementary Figure 1. Phenotypic characterization and the functional profiling of iγδT cells differentiated from hiPSCs

A. A flow cytometry (FCM) analysis showing the percentage of CD3-positive iγδT cells differentiated from hiPSC clone 121-3.
B. FCM analysis showing the percentage of CD3-positive iγδT cells differentiated from the CAR-expressing γδT-iPSC line.
C. FCM analysis of the γδTCR expression in iγδ T cells and peripheral blood–derived γδ T cells (PBγδT cells). The iγδT cells exhibited a higher mean fluorescence intensity (MFI) with a single peak, in contrast to the bimodal distribution observed in the PBγδT cells.
D. Repertoire analysis of iγδT cells (derived from clone 121-3) at day 34 of differentiation. TCR repertoire profiling revealed predominant usage of TRGV9 and TRDV2. Amino acid sequence reads with a frequency of <1% were excluded, as such low-frequency reads may reflect sequencing errors.
E. FCM analysis of the CD4, CD8α, and CD8β expression in CD3-positive and CD3-negative iγδ T cells at the differentiation stage (day 26) and final product stage (day 58).
F. FCM analysis of the NKG2D, DNAM-1, and NKp30 expression in CD3-positive and CD3-negative iγδT cells at the final product stage, with >90% of the cells expressing these receptors.
G. FCM analysis of the CD45RA and CD27 expression in iγδ T cells, indicating a terminal effector phenotype (CD45RA+CD27-).
H. FCM analysis of the cytotoxic molecule expression. iγδ T cells were stimulated by co-culture with Jurkat cells at an E:T ratio of 2:1 for 6h in the presence of brefeldin A and produced IFN-γ , perforin, and granzyme B.

Figure S2

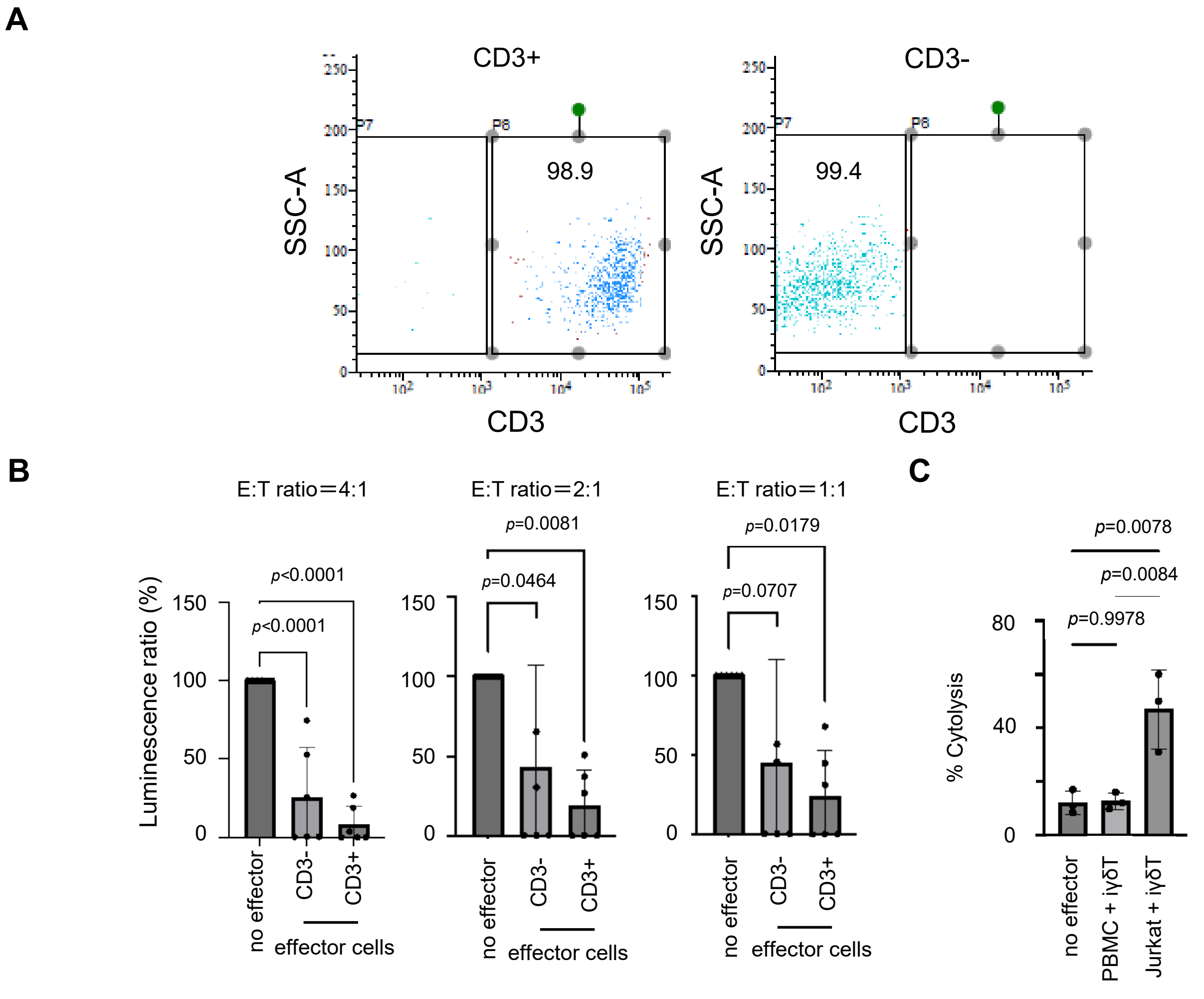

Supplementary Figure 2. Cytotoxic activity and safety profile of iγδT cells

A. A flow cytometry (FCM) analysis showing the sorting of iγδT cells into CD3-positive and CD3-negative cells.

B. The relative luminescence intensity of JurkatA containing CD3-positive cells and JurkatA containing CD3-negative cells, with the luminescence intensity of NC set to 100% (E:T ratios of 4:1, 2:1, and 1:1).

C. A mixed lymphocyte reaction (MLR) assay using PBMCs from three independent donors. The iγδT cells showed minimal cytotoxicity and a low alloreactivity toward PBMCs, whereas a robust cytotoxic activity was observed against Jurkat cells.

Figure S3

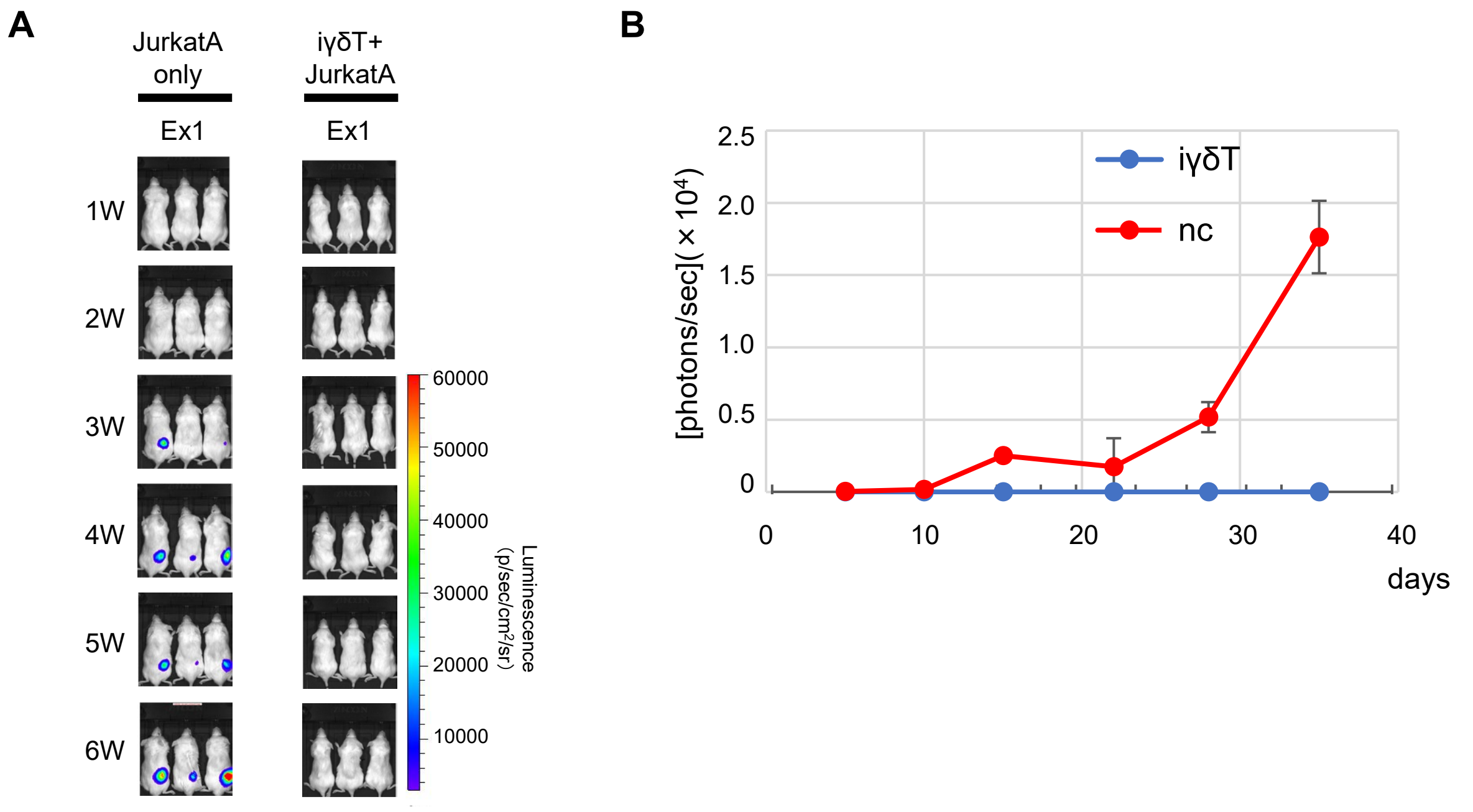

Supplementary Figure 3. In vivo antitumor activity of iγδT cells

A. Weekly bioluminescence images of the mice injected with JurkatA cells, with or without iγδT cell treatment, derived from a single differentiation batch (n = 3 per group) from the same lot (n = 3).

B. Quantification of the bioluminescence intensity of JurkatA tumors over time in mice. "nc" indicates"no effector control group." iγδT cell treatment resulted in a significant reduction in the tumor burden, with limited inter-animal variability.

Figure S4

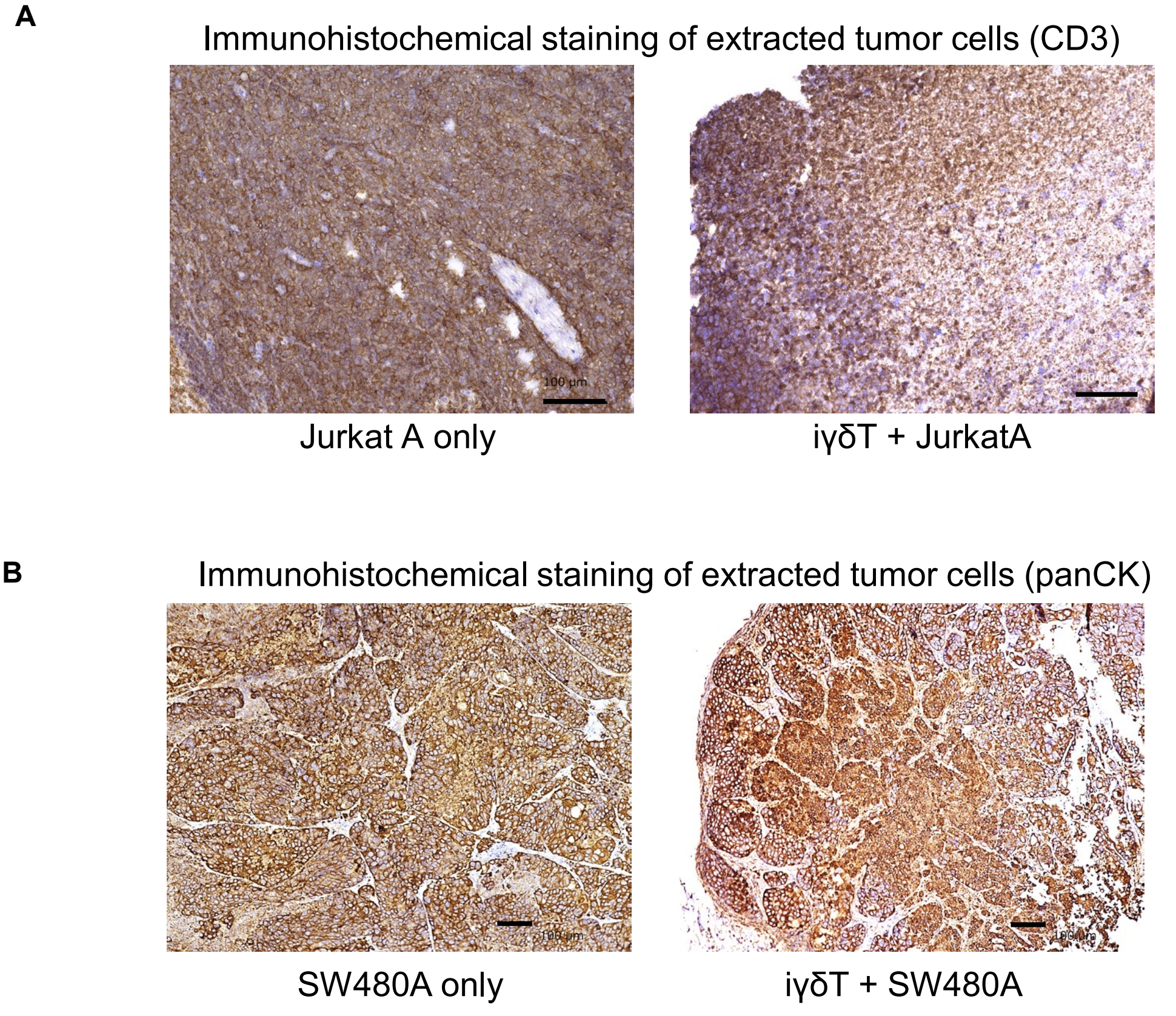

Supplementary Figure 4. Immunohistochemical Analysis of Tumor Tissues from JurkatA and SW480A Xenografts Following iγδT Cell Treatment

A. Left panel: CD3 immunohistochemical staining of tumor tissues extracted from JurkatA (NC group) transplanted into mice. Scale bars, 100 µm.

Right panel: CD3 immunohistochemical staining of tumor tissues extracted from JurkatA (iγδT group) transplanted into mice. Scale bars, 100 µm.

B. Left panel: panCK immunohistochemical staining of tumor tissues extracted from SW480A (NC-treated group) transplanted into mice. Scale bars, 100 µm.

Right panel: panCK immunohistochemical staining of tumor tissues extracted from SW480A (iγδT cell-treated group) transplanted into mice. Scale bars, 100 µm.

Figure S5

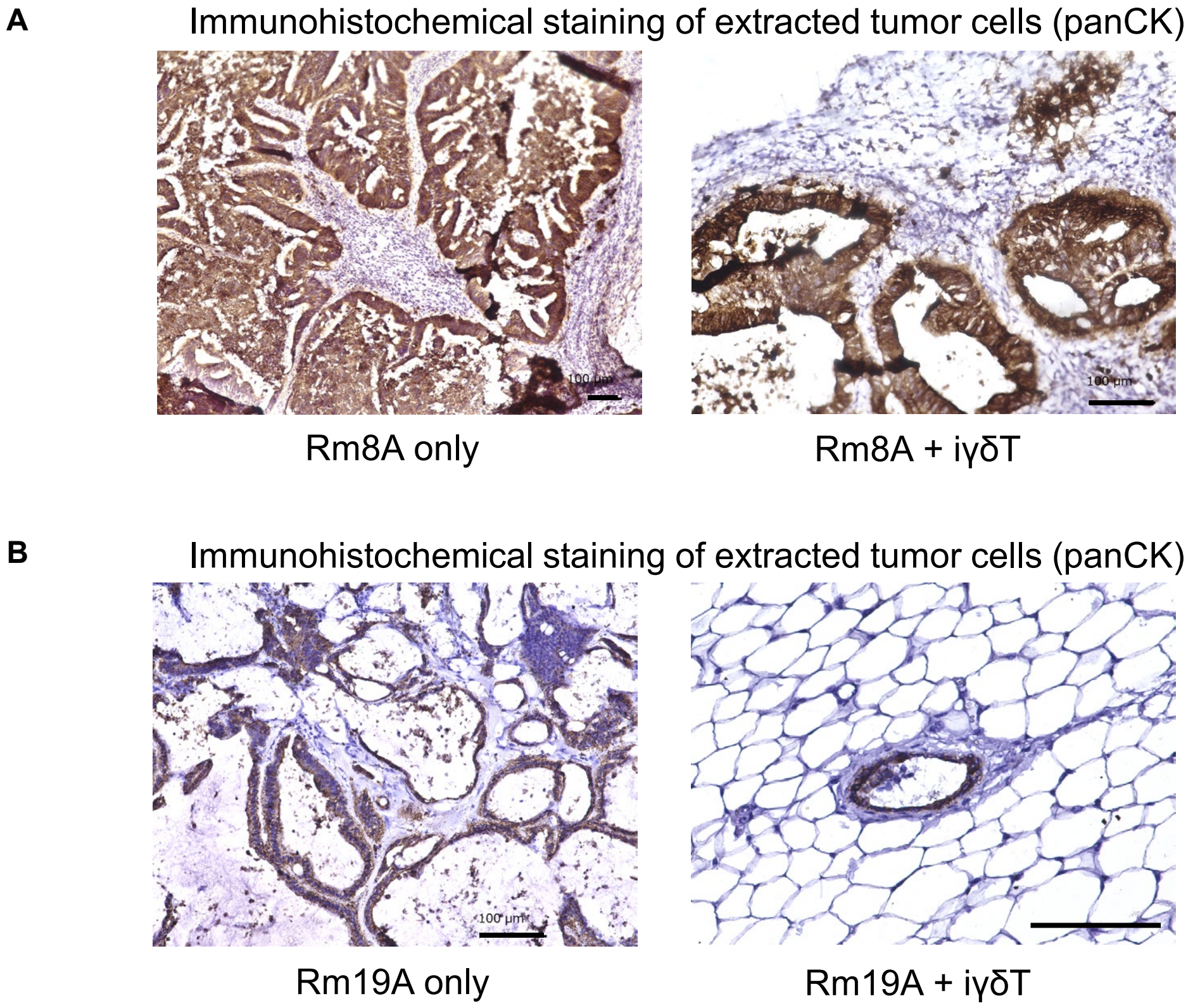

Supplementary Figure 5. panCK Immunohistochemical Staining of Rm8A and Rm19A Xenograft Tumors Following iγδT Cell Treatment

A. Left panel: panCK immunohistochemical staining of tumor tissues extracted from Rm8A (NC group) transplanted into mice. Scale bars, 100 µm.

Right panel: panCK immunohistochemical staining of tumor tissues extracted from Rm8A (iγδT cell locally treated group) transplanted into mice. Scale bars, 100 µm.

B. Left panel: panCK immunohistochemical staining of tumor tissues extracted from Rm19A (NC group) transplanted into mice. Scale bars, 100 µm.

Right panel: panCK immunohistochemical staining of tumor tissues extracted from Rm19A (iγδT cell-treated group) transplanted into mice. Scale bars, 50 µm.

Figure S6

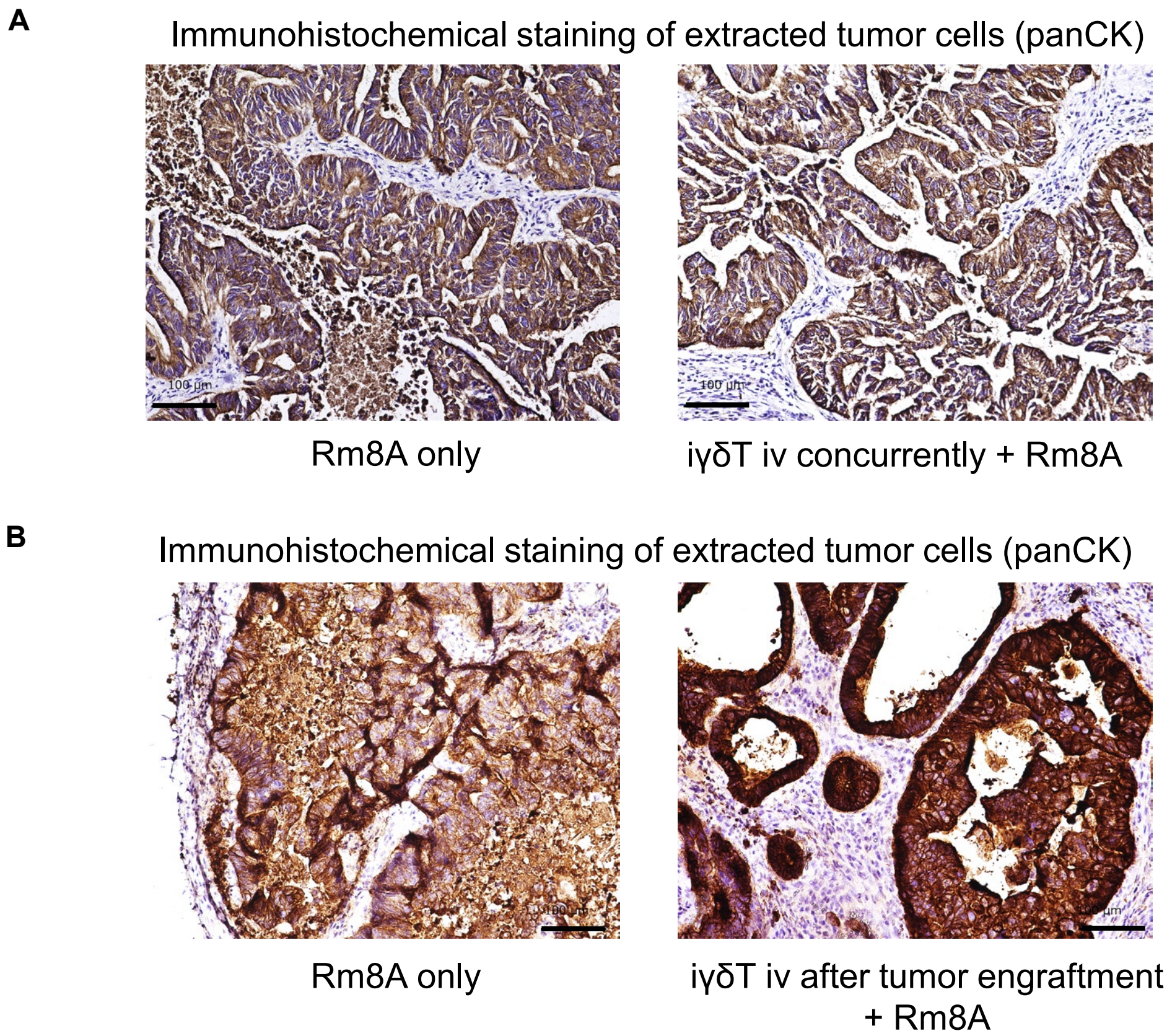

Supplementary Figure 6. panCK Immunohistochemical Staining of Rm8A Xenograft Tumors Following Intravenous iγδT Cell Administration

A. Left panel: panCK immunohistochemical staining of Rm8A (NC group) transplanted into mice. Scale bars, 100 µm.

Right panel: panCK immunohistochemical staining of Rm8A (iγδT cell intravenously co-administered group) transplanted into mice. Scale bars, 100 µm.

B. Left panel: panCK immunohistochemical staining of Rm8A (NC-treated group) transplanted into mice. Scale bars, 100 µm.

Right panel: panCK immunohistochemical staining of Rm8A (the group with iγδT cells intravenously administered after tumor engraftment) transplanted into mice. Scale bars, 100 µm.

Table S1

rank	TRGV	TRGJ	CDRJ	%reads
1	TRDV2	TRDJ1	CACDPLLGDTPRYTDKLIF	90.31884
2	TRDV2	TRDJ1	CACDPLLWDTPRYTDKLIF	2.062132
3	TRDV2	TRDJ1	CACDPLLVDTPRYTDKLIF	1.023523
4	TRDV2	TRDJ1	CACDPLLGDMPRYTDKLIF	0.327538
5	TRDV2	TRDJ1	CAFDPLLGDTPRYTDKLIF	0.31552
6	TRDV2	TRDJ1	CVCDPLLGDTPRYTDKLIF	0.282536
7	TRDV2	TRDJ1	CACDPLLGDTPRYTDKPIF	0.245717
8	TRDV2	TRDJ1	CAGDPLLGDTPRYTDKLIF	0.199437
9	TRDV2	TRDJ1	CACDPLLGETPRYTDKLIF	0.196369
10	TRDV2	TRDJ1	CACDPLLGDTPRYTEKLIF	0.178215
11	TRDV2	TRDJ1	CACDPLLGDTPRYTDKLTF	0.171312
12	TRDV2	TRDJ1	CACDPLLGDTPRYTNKLIF	0.170289
13	TRDV2	TRDJ1	CACDPLRGDTPRYTDKLIF	0.132703
14	TRDV2	TRDJ1	CACDPLLGDTPRDTDKLIF	0.130913
15	TRDV2	TRDJ1	CACDPLLGDTPRYTDKLIC	0.12989
16	TRDV2	TRDJ1	CACDPLLGDTPRYTDKLIV	0.126566
17	TRDV2	TRDJ1	CARDPLLGDTPRYTDKLIF	0.125543
18	TRDV2	TRDJ1	CACDPLLGDTPRYTDKLIL	0.117106
19	TRDV2	TRDJ1	WACDPLLGDTPRYTDKLIF	0.108924
20	TRDV2	TRDJ1	CACDPRLGDTPRYTDKLIF	0.104577
21	TRDV2	TRDJ1	GACDPLLGDTPRYTDKLIF	0.093838
22	TRDV2	TRDJ1	CTCDPLLGDTPRYTDKLIF	0.090258
23	TRDV2	TRDJ1	CACDLLLGDTPRYTDKLIF	0.087446
24	TRDV2	TRDJ1	CAWDPLLGDTPRYTDKLIF	0.086679
25	TRDV2	TRDJ1	CACDPLLGDTSRYTDKLIF	0.081821
26	TRDV2	TRDJ1	YACDPLLGDTPRYTDKLIF	0.077729
27	TRDV2	TRDJ1	CACDSLLGDTPRYTDKLIF	0.074661
28	TRDV2	TRDJ1	CACDPLLLDTPRYTDKLIF	0.072871
29	TRDV2	TRDJ1	CACYPLLGDTPRYTDKLIF	0.07057
30	TRDV2	TRDJ1	CACDPLLGDTPKYTDKLIF	0.066991
31	TRDV2	TRDJ1	CACDPLLGDTPRYIDKLIF	0.066479
32	TRDV2	TRDJ1	CACDPLLGDTLRYTDKLIF	0.065712
33	TRDV2	TRDJ1	CACDPLLGDTPGYTDKLIF	0.065201
34	TRDV2	TRDJ1	CAYDPLLGDTPRYTDKLIF	0.064434
35	TRDV2	TRDJ1	CACDPLLGGTPRYTDKLIF	0.061621
36	TRDV2	TRDJ1	CACGPLLGDTPRYTDKLIF	0.059576
37	TRDV2	TRDJ1	RACDPLLGDTPRYTDKLIF	0.05932
38	TRDV2	TRDJ1	CACDPLLGDTPRYTDKRIF	0.055996
39	TRDV2	TRDJ1	CACDPLLGDTPRYADKLIF	0.052928
40	TRDV2	TRDJ1	CASDPLLGDTPRYTDKLIF	0.052672
41	TRDV2	TRDJ1	CACDPLLGDTPRCTDKLIF	0.051649
42	TRDV2	TRDJ1	CACDPLLGDTPRYTDKFIF	0.051394
43	TRDV2	TRDJ1	CACDPLLGDAPRYTDKLIF	0.048325
44	TRDV2	TRDJ1	CACDPLLGDTPRYTDKLVF	0.043979
45	TRDV2	TRDJ1	CACDPLLGDTPRYTDKLSF	0.038353
46	TRDV2	TRDJ1	CACDPLLGDTPRYTDRLIF	0.034774
47	TRDV2	TRDJ1	CACDPLLGDTPRYTGKLIF	0.031705
48	TRDV2	TRDJ1	CACDPLLGNTPRYTDKLIF	0.021989
49	TRDV2	TRDJ1	CAFDPLLWDTPRYTDKLIF	0.021222
50	TRDV2	TRDJ1	CACDPPLGDTPRYTDKLIF	0.021222

rank	TRDV	TRDJ	CDRJ	%readas
1	TRGV9	TRGJP	CALWEVQELGKKIKVF	87.66021
2	TRGV9	TRGJP	CALWEVQELGKXIKVF	3.23425
3	TRGV9	TRGJP	CALWEVQELXKKIKVF	2.969439
4	TRGV9	TRGJP	YALWEVQELGKKIKVF	0.401824
5	TRGV9	TRGJP	CALLEVQELGKKIKVF	0.299832
6	TRGV9	TRGJP	CALWEVQELGKKINVF	0.288158
7	TRGV9	TRGJP	CALWEVQELDKKIKVF	0.262967
8	TRGV9	TRGJP	CVLWEVQELGKKIKVF	0.23962
9	TRGV9	TRGJP	CALWEVQELGKKIKIF	0.186166
10	TRGV9	TRGJP	CALWEVQELGKRIKVF	0.181865
11	TRGV9	TRGJP	CALWEVQELGKKIKLF	0.180636
12	TRGV9	TRGJP	CALWEVQELGKKIKVL	0.169577
13	TRGV9	TRGJP	CALGEVQELGKKIKVF	0.140085
14	TRGV9	TRGJP	CALCEVQELGKKIKVF	0.132712
15	TRGV9	TRGJP	CALWEGQELGKKIKVF	0.124111
16	TRGV9	TRGJP	CAVWEVQELGKKIKVF	0.121039
17	TRGV9	TRGJP	CAFWEVQELGKKIKVF	0.107522
18	TRGV9	TRGJP	CALWEVQEVGKKIKVF	0.103835
19	TRGV9	TRGJP	CALWEVQEWGKKIKVF	0.101992
20	TRGV9	TRGJP	CALWEVRELGKKIKVF	0.101378
21	TRGV9	TRGJP	CALWEVQELGKKIRVF	0.089089
22	TRGV9	TRGJP	CALWEVQELGKKIEVF	0.086017
23	TRGV9	TRGJP	CALWEVQELGKKSKVF	0.080488
24	TRGV9	TRGJP	CALWEVQELGKEIKVF	0.079259
25	TRGV9	TRGJP	GALWEVQELGKKIKVF	0.076801
26	TRGV9	TRGJP	RALWEVQELGKKIKVF	0.074344
27	TRGV9	TRGJP	CALWGVQELGKKIKVF	0.073729
28	TRGV9	TRGJP	CAWWEVQELGKKIKVF	0.070657
29	TRGV9	TRGJP	CALWEVQELGKKIKGF	0.068814
30	TRGV9	TRGJP	CSLWEVQELGKKIKVF	0.065742
31	TRGV9	TRGJP	CALWEVQELCKKIKVF	0.065127
32	TRGV9	TRGJP	CALWEVQELGKKIKVC	0.064513
33	TRGV9	TRGJP	CALWEVQELGRKIKVF	0.06267
34	TRGV9	TRGJP	CALWEVQGLGKKIKVF	0.061441
35	TRGV9	TRGJP	WALWEVQELGKKIKVF	0.058983
36	TRGV9	TRGJP	CALWEVQELGKKTKVF	0.057754
37	TRGV9	TRGJP	CALWEVQDLGKKIKVF	0.052225
38	TRGV8	TRGJ1	CATWDYYKKLF	0.052225
39	TRGV9	TRGJP	CTLWEVQELGKKIKVF	0.049153
40	TRGV9	TRGJP	CALWEVQELGKKVKVF	0.047924
41	TRGV9	TRGJP	CASWEVQELGKKIKVF	0.043623
42	TRGV9	TRGJP	CALWEVQELGKKIKAF	0.038708
43	TRGV9	TRGJP	CALWEVQELGKKIKVV	0.038093
44	TRGV9	TRGJP	SALWEVQELGKKIKVF	0.031949
45	TRGV9	TRGJP	CALWEVQELGKKIKVS	0.028263
46	TRGV9	TRGJP	FALWEVQELGKKIKVF	0.027034
47	TRGV9	TRGJP	CALWEVQELGEKIKVF	0.021504
48	TRGV5	TRGJ1	CATNYYKKLF	0.020276
49	TRGV9	TRGJP	CALWEVQELGKKITVF	0.019047
50	TRGV9	TRGJP	CALWEVHELGKKIKVF	0.017818

Table S1. Dominant TCR clonotypes identified in iγδT cells

TCR repertoire sequencing of day-34 iγδT cells derived from the 121-3 γδT-iPSC line. The table lists the top 50 TCRδ and TCRγ clonotypes ranked by read frequency. A single dominant TRDV2-TRDJ1 clonotype and a single dominant TRGV9-TRGJP clonotype represented the majority of reads, consistent with preservation of the parental Vγ9Vδ2 T-cell clonotype during differentiation.
